# Mass‐spectrometry analysis of the human pineal proteome during night and day and in autism

**DOI:** 10.1111/jpi.12713

**Published:** 2021-01-11

**Authors:** Guillaume Dumas, Hany Goubran‐Botros, Mariette Matondo, Cécile Pagan, Cyril Boulègue, Thibault Chaze, Julia Chamot‐Rooke, Erik Maronde, Thomas Bourgeron

**Affiliations:** ^1^ Human Genetics and Cognitive Functions Institut Pasteur UMR 3571 CNRS University Paris Diderot Paris France; ^2^ Precision Psychiatry and Social Physiology laboratory CHU Ste‐Justine Research Center Department of Psychiatry University of Montreal Quebec QC Canada; ^3^ Institut Pasteur Unité de Spectrométrie de Masse pour la Biologie (MSBio) Centre de Ressources et Recherches Technologiques (C2RT) USR 2000 CNRS Paris France; ^4^ Paris Descartes University Paris France; ^5^ Service de Biochimie et Biologie Moléculaire INSERM U942 Hôpital Lariboisière APHP Paris France; ^6^ Institute for Anatomy II Faculty of Medicine Goethe University Frankfurt Germany

**Keywords:** antioxidant, autism spectrum disorders, chaperon proteins, circadian rhythm, energy production, mass spectrometry, pineal gland

## Abstract

The human pineal gland regulates day‐night dynamics of multiple physiological processes, especially through the secretion of melatonin. Using mass‐spectrometry‐based proteomics and dedicated analysis tools, we identify proteins in the human pineal gland and analyze systematically their variation throughout the day and compare these changes in the pineal proteome between control specimens and donors diagnosed with autism. Results reveal diverse regulated clusters of proteins with, among others, catabolic carbohydrate process and cytoplasmic membrane‐bounded vesicle‐related proteins differing between day and night and/or control versus autism pineal glands. These data show novel and unexpected processes happening in the human pineal gland during the day/night rhythm as well as specific differences between autism donor pineal glands and those from controls.

## 1 INTRODUCTION

The pineal gland (also named Epiphysis cerebri) is a small endocrine gland located at the border between the mesencephalon and diencephalon of the brain.^1^, ^2^ Unlike much of the rest of the brain, the pineal gland is not isolated from the body by the blood‐brain barrier and belongs to the so‐called “circumventricular” organs.^3^, ^4^ It is highly vascularized and composed of a large and dense anastomosed network of capillaries.^3^, ^5^, ^6^ The main known function of the pineal gland is to produce the hormone melatonin.^7^ This key molecule derived from serotonin transfers the circadian and seasonal information from the “master clock” located in the suprachiasmatic nuclei (SCN) of the hypothalamus to many “slave clocks” through the body. Although thought to exert its physiological functions mostly through G‐protein‐coupled receptors,^8^ melatonin has also been described as an antioxidant molecule.^9^, ^10^

The production of melatonin in humans follows a circadian rhythm, which is synchronized to a 24‐h daily light‐dark cycle, with high nocturnal (night) and low diurnal (day) levels.^2^, ^11^, ^12^ In all mammalian species investigated so far, the dynamics in pineal melatonin synthesis is known to be dependent on the elevated release of the neurotransmitter norepinephrine (NE) from sympathetic nerve terminals originating in the superior cervical ganglion during nighttime. NE activates the 3′5′‐cyclic adenosine monophosphate (cAMP)‐signaling pathway in pinealocytes, ultimately leading to the acetylation of serotonin by the arylalkylamine N‐acetyltransferase (AANAT).^13^ Subsequently, N‐acetyl serotonin is converted into melatonin by the acetylserotonin‐methyltransferase (ASMT), formerly known as hydroxyindole O‐methyltransferase (HIOMT).^2^, ^14^, ^15^

The human pineal gland contains a follicular parenchyma consisting of pinealocytes, interstitial cells, nerve fibers, and capillaries. The interstitial cells contain filaments immunoreactive for GFA like the astrocytes in the brain.^16^ The pineal follicles are surrounded by septae of loose connective tissue with blood vessels, nerve fibers and some nerve cell bodies. Macrophages, mast cells and striated muscle fibers can be found in the septae of the human pineal. Finally, the human pineal is surrounded by a connective tissue capsule derived from the arachnoid. The main cell type of the mammalian pineal parenchymal is the pinealocyte, (~95%), however, many endothelial and vascular smooth muscle cells, few glia, phagocytic cells, and rarely neurons are present, too.^17^, ^18^ The synaptic ribbons are electron‐dense structures tethering synaptic vesicles, considered as specific marker structure for the pinealocytes with a unique type of chemical synapse, referred by Jouvet et al (1994) as the ribbon synapse.^19^, ^20^, ^21^, ^22^, ^23^ The ribbon synapse is not a classical synapse and its protein composition differs from conventional synapses reflecting different modes of transmitter release. In contrast to other synapses, there is no postsynaptic specialization in apposition to the ribbon and vesicles. The ribbon is often found in the cytoplasm of the pinealocyte away from the cell membrane. While mammalian pinealocytes are not light sensitive, they contain photoreceptor‐specific proteins such as the S‐antigen, rhodopsin, and recoverin.^17^, ^24^ Glial fibrillary acidic protein (GFAP) which is also found in the fibrillary astrocytes of the central nervous system, was observed in pineal interstitial cells.^25^

Melatonin secretion is under the control of adrenergic and peptidergic inputs regulating AA‐NAT activity. In addition to the sympathetic innervation, pineal gland receives peptidergic (eg, vasoactive intestinal peptide (VIP); peptide histidine isoleucine (PHI); neuropeptide Y (NPY), arginine vasopressin (AVP) fibers, playing a modulating role in pineal function. They are differently involved in the cAMP and calcium signaling.^26^, ^27^

Abnormal pineal gland and/or melatonin synthesis was observed in many human diseases, such as dementia,^28^ mood disorders,^29^ Alzheimer's disease,^30^, ^31^, ^32^ and in patients with Smith‐Magenis syndrome who display an inverted melatonin circadian rhythm and present severe autistic behavior.^33^, ^34^ Reduced melatonin levels have also been reported in individuals with autism and in circadian rhythm sleep disorders.^35^, ^36^, ^37^, ^38^, ^39^ The role of melatonin has also recently received attention in cancer,^40^ diabetes type 2,^41^ multiple sclerosis,^42^ and acute myocardial infarction.^43^

In the last decade, in order to identify genes that exhibit day/night differential expression, transcript analysis using cDNA microarray technologies revealed a list of genes that are expressed in the pineal gland of zebrafish, mice, rats and humans.^44^, ^45^, ^46^, ^47^, ^48^, ^49^ Some of these studies were performed under light‐dark (=“diurnal”) or constant (= “circadian”) cycles^44^, ^45^, ^47^, ^48^; or following NE stimulation.^49^ Beside transcription, studying protein expression is crucial to identify the main proteins networks and physiological processes involved in the function of the pineal gland.

In the present study, we used autoptic human pineal glands and large‐scale proteomic analysis‐based proteomics to better understand the physiology of the pineal gland. We first identified the proteins expressed in the pineal gland and then characterized the main biological pathways displaying a diurnal modulation. Finally, we conducted the same study using pineal glands from individuals with autism to detect differentially expressed proteins that could have a role in this condition.

## 2 MATERIALS AND METHODS

For a global description of the workflow, see Figure S1.

### 2.1 Post‐mortal samples and protein extraction

All post‐mortal samples were obtained from the Maryland Brain and Tissue Bank, from the Harvard Brain Tissue Resource Center, or from Goethe‐Universität Frankfurt Inst. für Zelluläre und Molekulare Anatomie (Table S1).

The small pieces of pineal glands were 40 to 200 mg in weight (median 150 mg). We had no information about how the pineal capsule has been removed and if the samples are more rostral and caudal parts. The influence of post‐mortal “laying” time on RNA and protein has been investigated before and the proteolytic degradation is standard.^14^ The samples were transferred to a pre‐chilled glass‐glass conical Potter grinder (with good compliance between mortar and pestle) for tissue homogenization, on ice. We added cold lysis buffer (8M Urea, 2M Thiourea (RPN6301; GE Healthcare Life Sciences), 50 mM DTT (Sigma; 646 583), 4% CHAPS 20 mM (GE Healthcare Life Sciences), Tris‐HCl pH 8.5 plus protease inhibitors (Sigma, P8340) and homogenized the tissue by manual up‐and‐down strokes and minimize foaming. We then transferred samples to microfuge tubes (samples are kept on ice at all times). Debris were removed by centrifugation of the homogenate at 15 ***g*** for 20 minutes at 4°C and the supernatant was transferred to a new tube. Aliquots of supernatants were immediately frozen in crushed carbon dioxide and stored at −80°C. A small amount of the supernatant was used for determination of total protein concentration using BCA protein test (Thermo Scientific).

### 2.2 Western blot assay

Western blot analyses were performed on autoptic human pineal glands using supernatants as described in the preparation of samples for MS. Equal amounts of protein extracts (50 µg) were resolved on a 4%‐12% precast gels under reducing conditions (NuPAGE) and electro‐transferred onto polyvinylidene difluoride membrane (iBlot 2 dry blotting system, Thermo Fisher Scientific) according to the manufacturer's protocol. The membranes were then blocked with 5% nonfat dry milk in PBS‐buffered saline containing 0.05% Tween 20 for 1 hour at room temperature and probed overnight at 4°C with anti‐AGRIN (sc‐374117) from Santa Cruz Biotechnology, Inc; and beta2‐microglobulin (#12851) from Cell Signaling Technology, Leiden, The Netherlands; anti‐agrin (GTX54904) GeneTex). Anti‐ß‐actin (A3854) Sigma‐Aldrich was used for normalization. After washing, the membranes were incubated for 1 hour at room temperature with secondary HRP conjugated antibody (#7076, #7074) and visualized using ECL detection kit (Thermo Scientific). The semi‐quantification densitometric analysis density of the specific bands was carried out using the ImageJ software to relatively quantify protein bands from western blot films. The quantification reflects the ratio of protein band relative to the housekeeping protein ß‐actin band. Statistical analyses showed a significantly higher AGRIN immunoreactivity in daytime pineal glands in comparison to nighttime. In contrast, ß2‐microglobulin showed a significantly higher immunoreactivity in nighttime in comparison to the daytime.

### 2.3 Protein digestion

Detergent was removed from the lysates and proteins were digested with Lys C and trypsin using the filter‐aided sample preparation (FASP) protocol (Wisniewski et al, 2009)^50^ using spin ultrafiltration units of nominal molecular weight cut of 30 000 Da. Briefly, to YM‐30 microcon filter units (Cat No. MRCF0R030, Millipore) containing protein concentrates, 200 μL of 8 mol/L urea in 0.1 mol/L Tris/HCl, pH 8.5 (UA), was added and samples were centrifuged at 14 000 ***g*** at 20°C for 40 minutes. This step was performed twice. Then 100 μL of 0.05 mol/L iodoacetamide in 8 mol/L urea was added to the filters and the samples were incubated in darkness for 20 min. Filters were washed twice with 200 μL of 8 mol/L UA. 40 µL of LysC in UA buffer was added to each sample and incubated overnight at 30°C. Finally, trypsin (Promega, Madison, WI) was added in 120 μL of 50 mM NH_4_HCO_3_ to each filter for 5h at 37°C. The protein to enzyme ratio was 100:1. Released peptides were collected by centrifugation and multiple rinse of the filter in 50 mM NH_4_HCO_3._ A last wash in 5M NaCl was performed to disrupt any adsorption of peptide to the cellulose membrane.

### 2.4 LC‐MS/MS analysis

Tryptic digests were analyzed by nanoLC‐MS/MS using an Ultimate 3000 RSLC system (Dionex, Thermo Scientific) coupled to the nanoflow electrospray ion source of a Q‐Exactive mass spectrometer (Thermo Scientific). Each digest was loaded on a trapping precolumn (C‐18 PepMap100, 5 µm, 100 Å, Dionex; Thermo Scientific,) at a flow rate of 10 µL/min of solvent A (2% Acetonitrile (ACN), 0.1% Formic Acid (FA)). Peptides were desalted on the precolumn for 2 minutes and separation was were performed using an in‐house 50 cm long column (C18 resin, Reprosil Pur, 1.9 µm, Dr Maish). Peptides were separated at a flow rate of 300 nL.min^‐1^ using a gradient of 2% to 55% solvent B (80% ACN, 0.1% FA) for 180 minutes, followed by a 10 minutes washing step at 98% solvent B and a reconditioning step at 2% B for 40 minutes.

Mass‐spectrometry acquisition was done using XCalibur (v 2.0) in a data‐dependent acquisition mode. Survey scan MS were acquired in the Orbitrap on the 300‐2000 m/z range with the resolution set to a value of 70 000 at m/z = 400 in profile mode (AGC target at 1E6). The 15 most intense ions per survey scan were selected for HCD fragmentation (NCE 27), and the resulting fragments were analyzed in the Orbitrap at 17 500 of resolution (m/z 400). Isolation of the parent ion was fixed at 2.5 m/z. Dynamic exclusion was employed within 20s. Data were acquired in profile mode.

### 2.5 Data analysis

Data were searched using MaxQuant (1.4.1.2 version) (with the Andromeda search engine) against the UniProt human database (2014.05.15, 88 473 entries).^51^ Proteins were identified by searching MS and MS/MS data of peptides against. Carbamidomethylation of cysteines was set as a fixed modification. Oxidation of methionine and protein N‐terminal acetylation were set as variable modifications. The minimum peptide length was specified to be 7 amino acids. The initial maximal mass tolerance in MS mode was set to 7 ppm, whereas fragment mass tolerance was set to 10 ppm for MS and MS/MS. Two peptides were required for protein identification and quantitation. The maximum false‐positive rate (FDR) for peptides and for proteins was specified as 0.01. Label‐free analysis was done by using the “match between run” feature of MaxQuant (2 minutes time window). LFQ data were used to perform statistical analysis. Figure S2 shows the Gaussian distribution of log‐normalized LFQ values in all measured pineal samples from non‐diagnosed control individuals. As expected, the majority of identified proteins are middle (25 < LFQ values > 30) or high abundant (2LFQ values > 30). Only a few proteins are very low abundant (10 < LFQ values > 15). Table S2 lists all the LFQ and Z‐score values with associated confidence interval.

### 2.6 Imputation of missing values

After log‐normalizing the LFQ values, we adopted a Monte‐Carlo approach to handle missing values. We run 2000 simulations in order to evaluate the confidence interval of the LFQ differences between the two groups of participants. For each simulation, we proceeded in these two steps:

● For each subject, we first computed an average LFQ for all proteins. To do this, we tested for each protein if both replicates were null. In this case, we marked the protein as missing and did not impute any value. If only one of the replicates was null, an imputed value was drawn from a normal distribution fitted on the mean and standard deviation of all the non‐null LFQs of the replicate. After imputation, we took the average of non‐missing replicates. We finally computed a Z‐score across all non‐missing values.
● For each protein, if both groups had less than 3 subjects with non‐missing value, the protein was marked as “Missing” for the overall experiment. If only one group had less than 3 subjects, we marked the protein as only present during the other condition (ie, “Only Day” or “Only Night”). Finally, if both groups had more than 3 subjects with non‐missing values (ie, with at least one replicate with a non‐null LFQ), the protein was considered as “Modulated” and we computed a student T‐test between the two groups. The observed T statistics and its p value were then stored.

Finally, based on all simulations, we computed a 95% confidence interval for all proteins marked as “Modulated” and considered the difference as statistically significant if the higher bound of p‐value interval was smaller than 0.05. We also rated the proteins marked as “Only day” and “Only night” with a confidence score:

$$\text{Confidence}=\frac{\frac{{\text{Nobs}}_{i}‐3}{{\text{Ntot}}_{i}‐3}‐\frac{{\text{Nobs}}_{j}}{2}}{\frac{{\text{Nobs}}_{i}‐3}{{\text{Ntot}}_{i}‐3}+\frac{{\text{Nobs}}_{j}}{2}}$$

where i and j stand for conditions, with i, j = Day, Night (resp. Night, Day) when the protein is marked as “Only day” (resp. “Only night”) (see Figure S9).

### 2.7 Detection of rhythmic protein abundance

In a complementary approach to the comparison between day and night, we also investigated the rhythmicity in protein abundance. We first used a standard chronobiology tool: the non‐parametric JTK‐cycle algorithm,^52^ which combines the Jonckheere‐Terpstra test for monotonic ordering and Kendall's τ test for association of measured quantities. We took advantage of the version 3.1 which considers missing values and replicates. Transcripts were considered circadian if JTK *P* < .01. For this significance, a FDR was calculated by permutations of the data and corresponded to 5%. To assess the stability of the detected cycling proteins, we used an alternative method from astrophysics, Generalized Lomb‐Scargle periodogram (GLS), which allows to detect periodic signals in unevenly sampled data^53^ To take into account the replicate and missing value, we used a Bayesian implementation of GLS (code available online here: https://github.com/mfouesneau/bgls).^54^

### 2.8 Gene ontology analyses

We identified enrichments of Genetic Ontology (GO) terms in gene sets related to the pineal gland itself, and on its states during day or night utilizing Cytoscape 3.3.0 with the plugin BINGO.^55^, ^56^ We used the genetic background consisting of all the proteins coding genes. We proceeded to a similar analysis with each subgroup of proteins (ie, Modulated, Only day, and Only night). To eliminate redundancy, we first filtered all the statistically significant Gene Ontology (GO) with less than 10 genes or more than 1000, and then used Enrichment Map to assess the graph of similarity between the different GO terms and to annotate the connected components (P‐value cutoff = 0.005; FDR Q‐value Cutoff = 0.01; Jaccard coefficient = 0.5).^57^

### 2.9 Protein‐protein interaction visualization

We concatenated five human interactome datasets provided online by the Dana Farber Cancer Institute: four high‐quality binary protein‐protein interactions (PPI) using a systematic primary yeast two‐hybrid assay (Y2H): HI‐I‐05,^58^ Venkatesan‐09,^59^ Yu‐11,^60^ and HI‐II‐14,^61^ plus one high‐quality binary literature dataset Lit‐BM‐13 comprising all PPI that are binary and supported by at least two traceable pieces of evidence (publications and/or methods).^61^ Proteins associated with, either statistical differences in intensity between day and night, or marked as only present during day or during the night, were then projected in the PPI network.

### 2.10 Data sharing statement

The mass‐spectrometry proteomics data have been deposited to the ProteomeXchange Consortium via the PRIDE partner repository with the dataset identifier PXD020501.^62^ All the rest of the data that supports the findings of this study are available in the supplementary material of this article.

## 3 RESULTS

### 3.1 Gene Ontology enrichment analysis of proteins from human pineal glands

**FIGURE 1:**
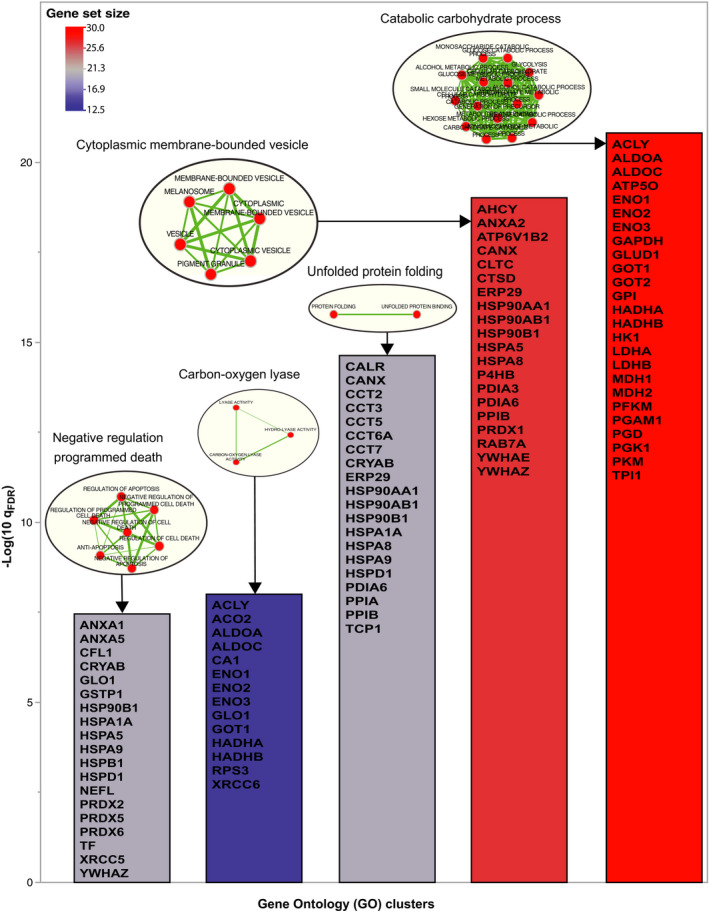
Biological functions of the most abundant proteins in the human pineal gland. Five first Gene Ontology (GO) clusters associated to the proteins with LFQ *Z*‐score > 1. GO with < 10 or > 1000 proteins were excluded from the BINGO results before running the Enrichment Map method. The color code represents the gene size of the cluster

The protein composition of the pineal gland was examined through Gene Ontology (GO) analysis. We used two computational approaches in this enrichment analysis. First, we included all the proteins detected by mass spectrometry (Non Zero; ie, proteins with LFQ > 0) and used the whole proteome as the background. Using this approach, 51 highly enriched pathways are detected (*q* value < 0.01; Figure S3; Table S3). Examples of seven relevant pathways according to their function in the pineal gland are: mitochondrial envelope, pigment granule, oxidation reduction, cytoplasmic membrane‐bounded vesicle, interspecies interaction organisms, oxidoreductase CH‐OH group donors, protein ubiquitination positive regulation. Then we used a more stringent filtering keeping only the most abundant proteins detected in the human pineal glands (1SD; ie, proteins with LFQ *Z*‐score > 1, see material and methods). Using this second approach, we identified five enriched GO clusters (Figure 1): catabolic carbohydrate process (GPI, TPI1, MDH1, MDH2, PGAM1, ENO1, ENO2, ENO3, HK1, LDHB, LDHA, PKM, PGK1, ALDOC, ALDOA, GAPDH, PFKM, PGD, ATP5O, GOT1, GOT2, HADHB, ACLY, GLUD1, HADHA), unfolded protein folding (ERP29, CCT5, HSPA1A, PPIB, CRYAB, HSPA8, HSP90AA1, HSP90AB1, HSPA9, CCT3, CCT2, HSPD1, PDIA6, CCT6A, TCP1, HSP90B1, CALR, CCT7, CANX, PPIA), cytoplasmic membrane‐bounded vesicle (ERP29, ATP6V1B2, P4HB, PPIB, CTSD, RAB7A, PDIA3, YWHAE, HSPA8, HSP90AA1, HSP90AB1, AHCY, HSPA5, ANXA2, CLTC, PDIA6, YWHAZ, HSP90B1, PRDX1, CANX), carbon‐oxygen lyase (GLO1, ENO1, ENO2, ENO3, RPS3, GOT1, ALDOC, XRCC6, ALDOA, CA1, ACO2, HADHB, ACLY, HADHA) and negative regulation of programmed death (GSTP1, HSPA1A, GLO1, PRDX6, PRDX5, TF, HSPB1, CRYAB, PRDX2, CFL1, NEFL, HSPA9, HSPA5, ANXA5, HSPD1, YWHAZ, HSP90B1, ANXA1, XRCC5). In addition to these enrichment analyses, proteins related to the melatonin synthesis cascade were also detected. This includes for example ASMT, the adrenergic receptor complex, G‐proteins, adenylate cyclase and the cAMP‐dependent protein kinase subunits. Interestingly, no peptide fragments of the ß‐adrenergic receptor or AANAT, the rate‐limiting enzyme of melatonin synthesis were detected.

### 3.2 Protein‐protein interaction network analysis of the human pineal glands

**FIGURE 2:**
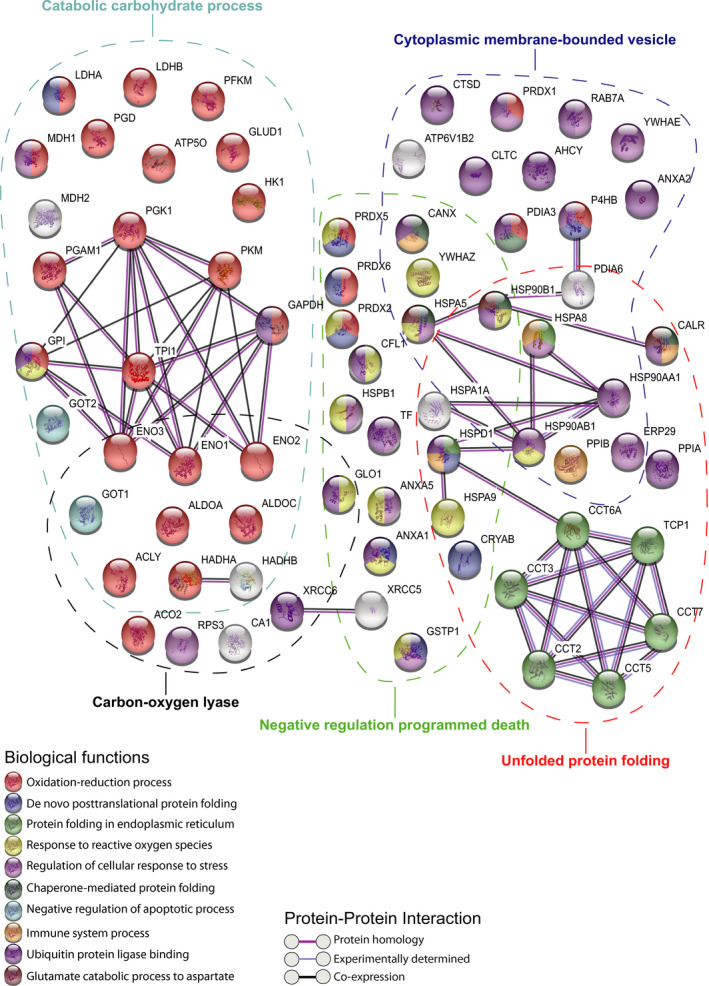
Core biological pathways of the human pineal gland. Enriched proteins (with 1SD) network analysis using the STRING software. The connecting lines between protein nodes indicate protein‐protein interactions (PPI). The five clusters of Figure 1 are circled in dashed color lines

To study the signaling pathways associated with the function of the pineal gland, we then analyzed the protein‐protein interaction (PPI) network of the proteins from the five top GO clusters using the STRING PPI network (Figure 2). Three different highly interconnected networks are depicted including the chaperonin‐containing TCP1 complex (CCT) proteins (CCT2, CCT3, CCT5, CCT6A, CCT7, TCP1), which assist the folding of nascent polypeptides and require ATP for their function. The second network includes members of the heat shock protein family (HSPA5, HSP90B1, HSPA8, HSP90AA1, HSP90AB1, HSPD1, HSPA1A, HSPA9) whose main role is to facilitate folding of proteins. Most of them are stress‐inducible proteins playing important roles when cells are exposed to stressful conditions. A third network includes proteins of cytoplasmic membrane‐bounded vesicles (PGK1, PKM, GAPDH, ENO2, ENO1, ENO3, GOT2, GPI, TPI1, PGAM1), which are involved in processes such as glycolysis and gluconeogenesis playing crucial roles in energy supply. Some proteins are included in overlapping pathways such as catabolic carbohydrate process and catalytic function such as carbon‐oxygen lyase (ENO1, ENO2, ENO3, ALDOA, ALDOC, GOT1, HADHA, HADHB, ACLY), cytoplasmic membrane‐bounded vesicle and unfolded protein folding (ERP29, HSP90AA1, HSP90AB1, PDIA6, PPIB, HSPA8, HSP90B1, CANX), cytoplasmic membrane‐bounded vesicle and negative regulation programmed death (HSPA5, YWHAZ), unfolded protein folding and negative regulation programmed death (HSPA1A, CRYAB, HSPA9, HSPD1) or carbon‐oxygen lyase and negative regulation programmed death (GLO1). Other small networks are present including two interacting DNA repair proteins XRCC5 and XRCC6 and two proteins HADHA and HADHB from the mitochondrial trifunctional protein complex that catalyzes the beta‐oxidation of fatty acids (we also detected other member of the fatty acid β‐oxidation cycle with LFQ > 25: ACAA2, ACAD9, ACADM, ACADVL, ACAT1, and ECHS1). Finally, peroxiredoxins (PRDX2, PRDX5, and PRDX6) are members of the peroxiredoxin family, a highly conserved family of thiol‐specific antioxidant enzymes protecting cells against injury associated with oxidative stress (for review: Hall A et al, 2009).^63^ They contribute to the regulation of a multitude of signaling pathways and several diseases including carcinogenesis, inflammation, type 2 diabetes, ocular oxidative damage (for review: Arevalo et al, 2018).^64^

### 3.3 Detection of proteins with a diurnal or nocturnal profile of expression

**FIGURE 3:**
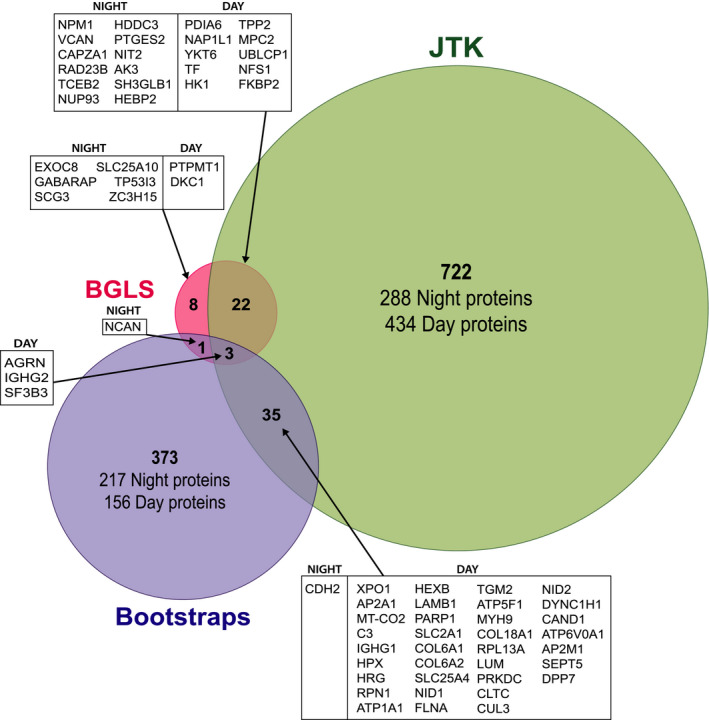
Diurnally modulated proteins in the human pineal gland. Three computational methods were used to identify regulated proteins. The Venn diagram represents the overlap proteins identified across those methods with areas of the circles and their overlap correlated to the corresponding numbers

Three computational methods were used to identify proteins that were differentially regulated during night and day (Figure 3). Since the Jonckheere‐Terpstra‐Kendall (JTK) method can detect any diurnal cycle and not only those aligned perfectly with day light, it allowed the identification of a higher number of modulated proteins (N = 782). The Bayesian generalized Lomb‐Scargle (BGLS) approach (see methods) was restricted to periods between 23 and 24 hours and identified 34 proteins. Finally, the bootstrap approach comparing abundance of proteins during day and night identified 451 proteins. Proteins identified by more than one approach are highlighted in the Venn diagram (Figure 3; Table S4). Rhythmic patterns of proteins exhibiting day (eg, AGRN and IGHG2) and night (eg, GFAP and CDH2) profiles are plotted in a panel (Figure S4) and a subset of modulated proteins were validated by Western blot (eg, GFAP B2M; Figure S5).

### 3.4 Protein‐protein interactions during night and day

**FIGURE 4:**
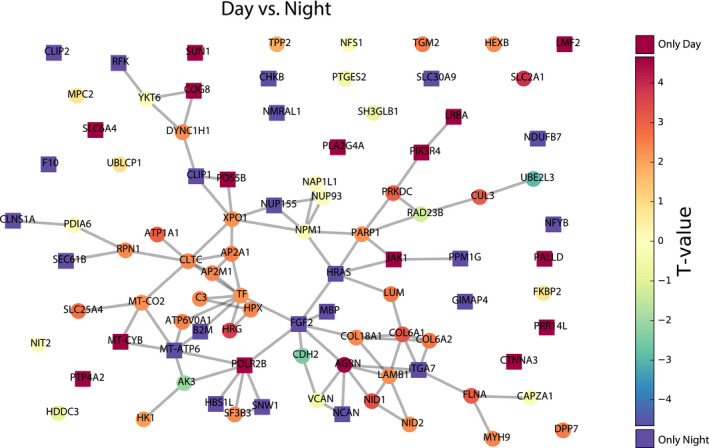
Interaction between the proteins modulated between day and night (for control participants only). The figure represents the protein‐protein interaction network of the most modulated proteins between day and night. Proteins that are more abundant during day or night are respectively in red or blue. While modulated proteins are represented by circles, those only observed during day or night are represented by squares

The PPI network of the most modulated proteins between day and night is presented in Figure 4. Fifteen proteins were detected only in the daytime pineal gland (LRBA, PIK3R4, JAK1, SUN1, PALLD, LMF2, SLC6A4, PDS5B, COG8, PLA2G4A, MT‐CYB, CTNNA3, POLR2B, PTP4A2, PRR14L), 23 proteins were only detected during nighttime (CHKB, CLIP2, NDUFB7, RFK, CLNS1A, SEC61B, B2M, SNW1, SLC30A9, PPM1G, HBS1L, MT‐ATP6, HRAS, NFYB, NCAN, FGF2, MBP, NMRAL1, CLIP1, NUP155, ITGA7, F10, GIMAP4) and 33 proteins were found oscillating between day and night periods. Agrin (AGRN) is the most highly cycling protein exhibiting upregulation during the day, followed by SLC2A1, HRG, then ATP1A1, NID1, COL6A1, PRKDC, CUL3, FLNA, SLC25A4 and finally DPP7, LUM, COL6A2, TGM2, MYH9, AP2M1, AP2A1, HPX, NID2, HEXB, DYNC1H1, RPN1, CLTC, XPO1, MT‐CO2, ATP6V0A1, PARP1, C3, LAMB1, COL18A1, SF3B3. Using the same approach, two proteins, CDH2 and UBE2L3 are upregulated during the night. As depicted in Figure 4, the majority of these proteins (70%) are interlinked forming a large interaction network composed of 44 protein nodes and 58 edges, where 71% are daytime proteins and 30% are nighttime proteins. In addition, two small networks (C3, HPX, HRG) and (CUL3, UBE2L3) were detected. CDH2 is linked to the large network via FGF2 while the other nighttime UB2L3 is linked to a daytime protein, CUL3. Other proteins displayed no close interaction in our construction (10 nighttime and 11 daytime).

### 3.5 The human pineal gland of individuals with autism

We reported previously that a subgroup of individuals with autism suffered from melatonin deficiency, which may exacerbate autistic traits and could increase the risk of sleep/circadian problems often reported in autism.^37^ Here we investigated the protein content of pineal glands from 7 patients with autism (Table S1) and compared results with proteins identified in controls. A total of 101 proteins were found statistically more abundant in non‐diagnosed controls—with 80 not even observed in individuals with autism—and 78 were more abundant in autism individuals (Table S5). Interestingly, there were 80 proteins observed only in non‐diagnosed controls, but none observed only in autism individuals.

**FIGURE 5:**
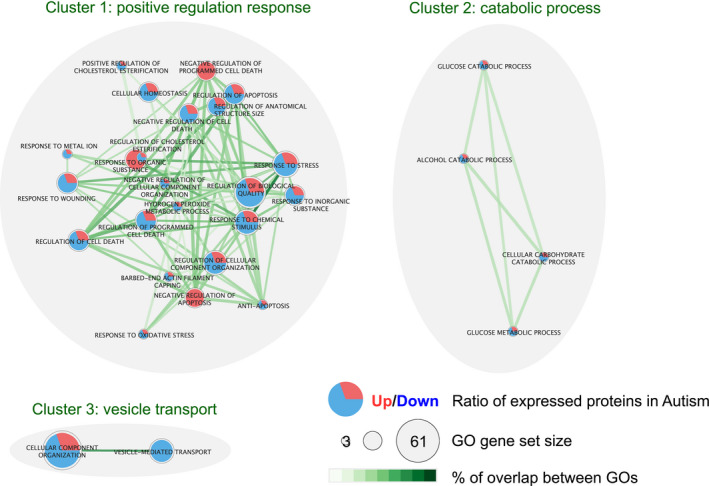
Enrichment map of autism associated proteins in the pineal gland. Circles represent enriched GO terms with fraction of up‐/down‐expressed proteins. Links represent proportion of shared proteins between GO terms. The complete list of proteins is available in Table S5

Enrichment analyses showed that three types of biological processes are modified in the autism group compared to the non‐diagnosed control group (Figure 5; for detailed PPI see Figure S6): the cellular response to stress with especially the regulation of apoptosis, catabolic processes associated with glucose and alcohol, and vesicle transport. While most of the associated biological pathways were downregulated in autism, interestingly some proteins with a higher expression in the autism group were also known to be involved in those processes (eg, inhibition of apoptosis).

Among the proteins detected with a higher abundance in the non‐diagnosed controls, 15 were listed in the SFARI database that lists genes/proteins associated with autism. Among them, CNTNAP2, a cell adhesion molecule associated with autism is observed in 7 of the 18 non‐diagnosed control pineal glands and absent of the 7 pineal glands from the individuals with autism. A complete list of the proteins displaying different levels in individuals with autism and non‐diagnosed controls is available in Table S5. The pineal samples studied were previously used to measure ASMT enzyme activities.^65^ Surprisingly, we show here that despite the low ASMT enzyme activity reported previously (Figure S7), the majority of the individuals with autism and ASMT enzyme deficiency have normal levels of the ASMT protein, suggesting that the quantity of ASMT is not the cause of the enzyme and melatonin deficiency in these patients.

## 4 DISCUSSION

### 4.1 A possible rhythmic metabolic influence on energy production in the human pineal gland

Our enrichment analyses identified several proteins in metabolic pathways such as the glycolysis, the Krebs cycle, the pentose phosphate pathway, and fatty acid beta‐oxidation. We observed, among others, the presence of non‐oscillating proteins including GPI, PGK, PKM, TPI1, ALDOC, and PGAM1 of the glycolysis pathway, MDH1 of the Krebs cycle,^66^ and HADHA and HADHB from fatty acid beta‐oxidation. More interestingly, we also observed that several of the abundant metabolic proteins were upregulated during nighttime including ENO1, LDHB, 6PGD and ATP5O. Enolases are carbon‐oxygen lyases involved both in glycolysis and gluconeogenesis and playing crucial roles in the ATP supply.^67^ Consistent with our result, ENO1 was previously shown to be upregulated in the rat pineal gland during night.^68^ LDH is the terminal enzyme of anaerobic glycolysis and involved in recycling of NADH to NAD^+^,^69^ 6PGD is involved in the pentose phosphate pathway generating NADPH whose function is critical to maintain redox balance under stress situations,^70^ and ATP5O a subunit of mitochondrial ATP synthase involved in ATP production within the mitochondrial respiratory chain.^71^ In this line, previous studies indicate a higher level of mitochondrial respiratory chain enzymes during the night.^72^ However we also identified several glycolytic enzymes which are upregulated during daytime including PFKM, GAPDH and HK1 indicating that they may influence several metabolic activities in the pineal gland in daytime. Altogether, these results suggest that energy production within the pineal gland is fine‐tuned during night and day. The nocturnal upregulation of some of these proteins suggest an increase of ATP production during the night that could be required for the nocturnal function of the pineal gland including melatonin synthesis.

### 4.2 The pineal gland as a source of antioxidant and chaperone proteins

There is a large body of evidence for a role of the pineal gland in antioxidative and inflammatory mechanisms. Melatonin itself has been reported as an anti‐inflammatory and antioxidative agent.^73^, ^74^ Analysis of our data also reveals the presence of enzymes, which are involved in protecting cells from ROS damaging effects such as superoxide dismutase, catalase, glutathione peroxidase, and peroxiredoxins.

Several proteins involved in glycolysis also possess antioxidant properties including 6PGDH, NADPH, HKI, the glutamate oxaloacetate transaminases (GOTs) involved in the production of the antioxidant NADPH,^75^ and GLO1, which was reported to be involved in antioxidative metabolism.^76^ These enzymes prevent overproduction of ROS and thereby prevent cell death.^77^, ^78^

We also identified several acute‐phase proteins (APPs) that are proteins that change their serum concentration in response to inflammation. It includes here haptoglobin, hemopexin, alpha‐2‐macroglobulin, alpha‐1‐antichymotrypsin, alpha‐1‐antitrypsin and haptoglobin‐ related protein. We do not assume that the pinealocytes themselves are secreting these factors, it is far more probable that the “pineal interstitial cells” of the pineal gland are the source of these factors. However, no matter which cell type(s) produces the APPs our data point to a role of the pineal gland as one source of the acute‐phase “secretome” in reaction to inflammation. We could detect chymase and cathepsin G suggesting the presence of mast cells that play important roles in inflammation and host defense,^79^ and were reported to be implicated in increased pineal calcifications.^80^

We also identified several chaperones such as the protein disulfide isomerase (PDI) family largely expressed in the endoplasmic reticulum (ER),^81^ and heat shock proteins (HSPs), which act as molecular chaperones in conditions of stress including carcinogenesis.^82^, ^83^

Most PDIs have an oxido‐reductase activity and function as molecular chaperones for correct protein folding by catalyzing the formation, cleavage, and rearrangement of the disulfide bonds in unfolded or misfolded proteins. For example, the ER resident protein 29 (ERP29) plays an important role in the processing of secretory proteins within the ER.^84^ The protein disulfide isomerase A3 (PDIA3) and protein disulfide isomerase A6 (PDIA6) mediate protein folding and also functions as a molecular chaperone that prevents the formation of protein aggregates. In interaction with calnexin (CANX), also an abundant protein of the human pineal gland, they modulate the folding of newly synthesized glycoproteins.

The main members of the HSPs detected in the human pineal glands include HSPA1A, HSPA5, HSPA8, HSPA9, HSPB1, HSPD1, HSP90B1, HSP90AA1 and HSP90AB1. These proteins most likely provide protection against stress by their role on nascent peptide chain synthesis, on proper peptide folding and elimination of misfolded protein.^85^

In addition to PDI and HSPs, we also detected members of the peroxiredoxin family of antioxidant enzymes (PRDX5 and PRDX6), which play a key role in response to increased ROS levels (Perkins, A et al, 2015) and of a particular family of chaperones, known as CCT: chaperonin‐containing tailless complex polypeptide 1 (TCP1) including CCT2, CCT3, CCT5, CCT7, CCT6A, and TCP1.^86^, ^87^ The proteins assist the folding of a nascent polypeptide and require ATP for their function.

Interestingly, several of these proteins involved in protein folding and chaperone function display a significant circadian oscillation in the human pineal gland. For example, HK1, GLO1, PDIA6, TF and HSPB1 display upregulation in daytime. In contrast, PRDX5 and PRDX6, are upregulated during nighttime. These results indicate that the human pineal gland possesses an elaborated antioxidant and chaperone machinery for the protection against oxidative stress and protein misfolding. Some members of these biological pathways seem to display circadian regulation, which might be essential to modulate the homeostasis of the pineal gland during night and day. While different antioxidants peak in the day, others, such as melatonin, peak at night.

### 4.3 Proteins regulating melatonin synthesis in the human pineal gland

Among the most important proteins known to be found in a pineal gland are the last two enzymes in the melatonin synthesis cascade, AANAT and ASMT (HIOMT).^13^, ^15^, ^88^ Interestingly neither our study, nor a recently published human pineal proteome study found peptide fragments corresponding to AANAT.^89^ ASMT, on the other hand and interestingly ASMTL, an ASMT‐like protein with a broader expression spectrum than ASMT, were identified in both studies. Moreover, tryptophan hydroxylase isozyme type I (TPH1) and aromatic‐amino‐acid decarboxylase (AADC), enzymes catalyzing important steps from the aromatic amino acid tryptophan to serotonin were also detected. Thus, all components necessary to synthesize melatonin from tryptophan are found in our human pineal proteome dataset with the exception of AANAT. One possible reason for the absence of AANAT may be its very short half‐life of about 8 minutes (in rodents) where it is stabilized to some extent by threonine‐31‐phosphorylation and 14‐3‐3 binding. However, both AANAT enzyme activity and melatonin content were measurable by biochemical assays and shown to be elevated at night in human,^14^, ^24^, ^90^, ^91^ so that so far, no explanation for these findings can be provided. Besides being ubiquitous proteins essential for many general biochemical processes, 14‐3‐3 proteins have been shown to heterodimerize with AANAT thus being essential for its activity and stability.^88^, ^92^, ^93^ In the present human proteome study and a previously published dataset,^89^ most known forms of 14‐3‐3 proteins were found to be present in the pineal gland (Figure S7). Remarkably, we have previously shown that ASMT enzyme activity was cycling between night (high) and day (low). Here, in contrast, the level of ASMT protein was stable during night and day (Figure S8). This suggests that the regulation of the enzyme activity is not caused by a difference in protein level, but most probably by other post‐translational mechanisms such as phosphorylation or dimerization. Interestingly, this mechanism might be dysregulated in a subset of individuals with autism (see below).

In addition to the enzymes required to synthesize melatonin, we detected the presence of many key components for the induction of melatonin synthesis.^94^, ^95^, ^96^ The dominance of PKA type II alpha and beta in pinealocytes has been confirmed for rat and cow.^97^, ^98^ The human pineal proteome data provided here confirm that PKA regulatory subunit type II alpha and beta are also the dominant subunits in the human pineal specimen investigated. This is further substantiated by the presence of the PKA type II‐binding A‐Kinase‐anchoring protein (AKAP) subtypes 8‐like, 9, 10, 11, 12 and 13, the transcriptional co‐activator CRTC‐1, the adenylate cyclase‐associated proteins CAP‐1 and −2, ß‐arrestin, phosducin, phosducin‐like protein and phosducin‐like protein‐3, other components of this pathway.

Parallel to the cAMP‐dependent pathway outlined above, adrenergic receptors in the pineal gland have been shown to activate protein‐kinase‐C and mitogen‐activated protein kinase pathways of which many different, previously described components have been identified here. Finally, all protein‐phosphorylation pathways leading to phospho‐proteins demand regulated protein‐phosphatases of which many were found in the present study, to name just one PP‐1a, which has been shown to be involved in the dephosphorylation of the serine‐133 phosphorylated form of the transcription factor CREB.^97^

The mechanisms by which the pineal gland synthesizes melatonin during the night are well documented,^1^, ^99^ but during the day, the mechanisms inhibiting the level of melatonin are less characterized. It was proposed that during the day acetylcholine (ACh) signals from the parasympathetic innervation inhibit the diurnal activity of AANAT and terminate melatonin production. There is also evidence for a cAMP‐independent inhibitory effect of glutamate on melatonin production.^100^ During the day, ACh activates nicotinic cholinergic receptors (nAChR) of pinealocytes resulting in membrane depolarization, opening voltage‐gated Ca^2^ ^+^ channels (VGCC) with a subsequent increase in the intracellular Ca^2+^ which ultimately triggers a release of glutamate resulting in an autocrine glutamatergic signal.^100^, ^101^, ^102^ Binding of glutamate to the metabotropic type 3 glutamate receptors (mGluR3) decreases cAMP levels resulting in decreased AANAT activity and melatonin synthesis.^103^, ^104^ However, most of these studies have been performed in rodents, therefore the impact of such mechanisms on pineal melatonin synthesis remains to be determined in humans.

Here, we observed that AGRN is a highly cycling protein in human pineal glands and primarily present at daytime. AGRN is a heparan sulfate proteoglycan originally discovered because of its ability to trigger the aggregation of ACh receptors (AChR) at the neuromuscular junction (NMJ).^105^, ^106^ AGRN is either secreted in the basal lamina of various tissues including the pineal gland or at the membranes of neurons and glial cells.^107^, ^108^, ^109^ As observed in the rat pineal gland,^112^, ^113^, ^114^ we also detected the AGRN receptor, the Na^+^/K^+^‐ATPase ion pumps (ATP1A3) in the human pineal gland, but without apparent circadian modulation. This receptor plays a key role in establishing the resting membrane voltage in neurons. AGRN inhibits the ATP1A3 pump activity resulting in membrane depolarization and increased action potential frequency.^115^

Altogether, in light with our observations, we can propose a melatonin inhibitory pathway where AGRN, mainly expressed in daytime, through interaction with its receptor ATP1A3, together with ACh, acts as a day signal to terminate the night actions of NE and sharpening the night‐to‐day transition. Taking into consideration the complexity of the processes and the myriad of proteins that could be involved, we cannot however determine which precise mechanisms actually take place and which specific pathways are involved.

### 4.4 The role of the human pineal gland in autism

We could analyze autoptic pineal glands from 7 individuals with autism. Autism is heterogeneous at the clinical and genetic levels and therefore this relatively small number of individuals precludes any generalization of our finding to autism in general. Nevertheless, melatonin production was repeatedly found deficient in a relatively large fraction of patients with autism and sleep problems are frequently reported.^65^, ^116^ We previously reported that taking as a threshold the 10th percentile of the control group, a large proportion (74%) of autistic patients displayed a reduced platelet ASMT activity,^37^, ^65^ but the mechanisms remain unknown. We also showed that the level of melatonin was highly inherited,^117^ but was not correlated to the size of the pineal gland.^118^ For a subset of patients, we could identify strongly deleterious ASMT mutations functionally associated with ASMT activity deficiency and low melatonin levels.^37^, ^91^, ^119^ But these rare mutations do not explain the deficit observed in a relatively large fraction of the individuals. Here, we show that autistic individuals with low ASMT activity display ASMT protein levels that are similar to the non‐diagnosed controls (Figure S7). Their low level of ASMT enzymatic activity is therefore not the consequence of a low level of ASMT protein. It is however reminiscent of the physiological reduction of ASMT activity during the day despite the stable ASMT protein over time. We could therefore hypothesize that the mechanism reducing ASMT activity during the day in controls is acting abnormally during the night in patients with autism leading to melatonin deficiency. Further work identifying the mechanisms of circadian regulation of ASMT enzyme activity might inform us on the melatonin deficiency in autism.

## 5 CONCLUSION AND PERSPECTIVES

Our proteomic analysis points at several metabolic enzymes, antioxidant, and chaperone proteins that are enriched in the human pineal glands. Some of the proteins seem to display a diurnal regulation suggesting that the biological functions associated with these proteins adapt to the physiological role of the pineal gland during night and day. Our study also provides a first characterization of the human pineal gland proteome in patients with autism indicating that the ASMT deficiency observed in a subset of patients is not always caused by a loss of ASMT protein. Further work is warranted to understand if the physiological downregulation of ASMT activity during the day is also abnormally active during the night leading to a severe melatonin deficit in a subset of individuals with autism. A larger sample size will also provide a clearer picture given the impact of age on sleep^120^ and the potential impact of the cause of death on the proteome.^121^ Finally, a comprehensive analysis of the protein partners and potential presence of post‐translational modifications (eg, phosphorylation, glycosylation, SUMOylation, ubiquitination) might also provide important clues to fully understand the circadian regulation of the human pineal gland.

## CONFLICT OF INTEREST

None of the authors has conflicts of interest pertaining to the present paper.

## AUTHOR CONTRIBUTIONS

Guillaume Dumas contributed to experimental design, analyses of the data, and drafting and reviewing the article. Hany Goubran‐Botros and Mariette Matondo contributed to experimental design, data collection, interpretation, and drafting and reviewing the manuscript. Cécile Pagan, Cyril Boulègue, and Thibault Chaze contributed to data collection. Julia Chamot‐Rooke, Erik Maronde, and Thomas Bourgeron participated in study design, coordination, and writing. All authors read and approved the final version.

## Supporting information

Fig S1

Fig S2

Fig S3

Fig S5

Fig S6

Fig S7

Fig S8

Fig S9

Table S1

Table S2

Table S3

Table S4

Table S5

Supplementary Material

## Data Availability

The mass spectrometry proteomics data have been deposited to the ProteomeXchange Consortium via the PRIDE partner repository with the dataset identifier PXD020501.
