# Supplementary material for: Mass‐spectrometry analysis of the human pineal proteome during night and day and in autism
Source: J Pineal Res. 2021 Jan 11;70(3):e12713. doi: 10.1111/jpi.12713 (PMC8047921; doi:10.1111/jpi.12713)
Supplement: Supplementary file 1 — Fig S1 [file JPI-70-e12713-s009.pdf]

Biological tissue samples: Pieces of autoptic human pineal glands

	ASD	Controls
Females	1	1
Males	6	17

Time of Death (t.o.d)*	ASD	Controls
Day (7am -18pm)	3	9
Night (18pm -7am)	4	8

**One control pineal gland without t.o.d*

↓

Sample preparation for mass spectrometry

Protein extraction and quantification
Proteolytic digestion

↓

Liquid chromatography–tandem mass spectrometry analysis (LC-MS/MS)

↓

Statistical data analysis

Maxquant search
Label free quantification
Imputation of missing values (2000 simulations)
Detection of rhythmic protein abundance
Comparison Day versus Night

↓

Three different methods to extract the modulated genes

Significantly Modulated BGLS Method
Significantly Modulated JTK Method
Significantly Modulated Bootstraps Method
Confident Only Day-Night

↓

Gene Ontology analysis

↓

Protein-Protein Interaction visualization
