# Supplementary figures and images for: Mass‐spectrometry analysis of the human pineal proteome during night and day and in autism
Source: J Pineal Res. 2021 Jan 11;70(3):e12713. doi: 10.1111/jpi.12713 (PMC8047921; doi:10.1111/jpi.12713)

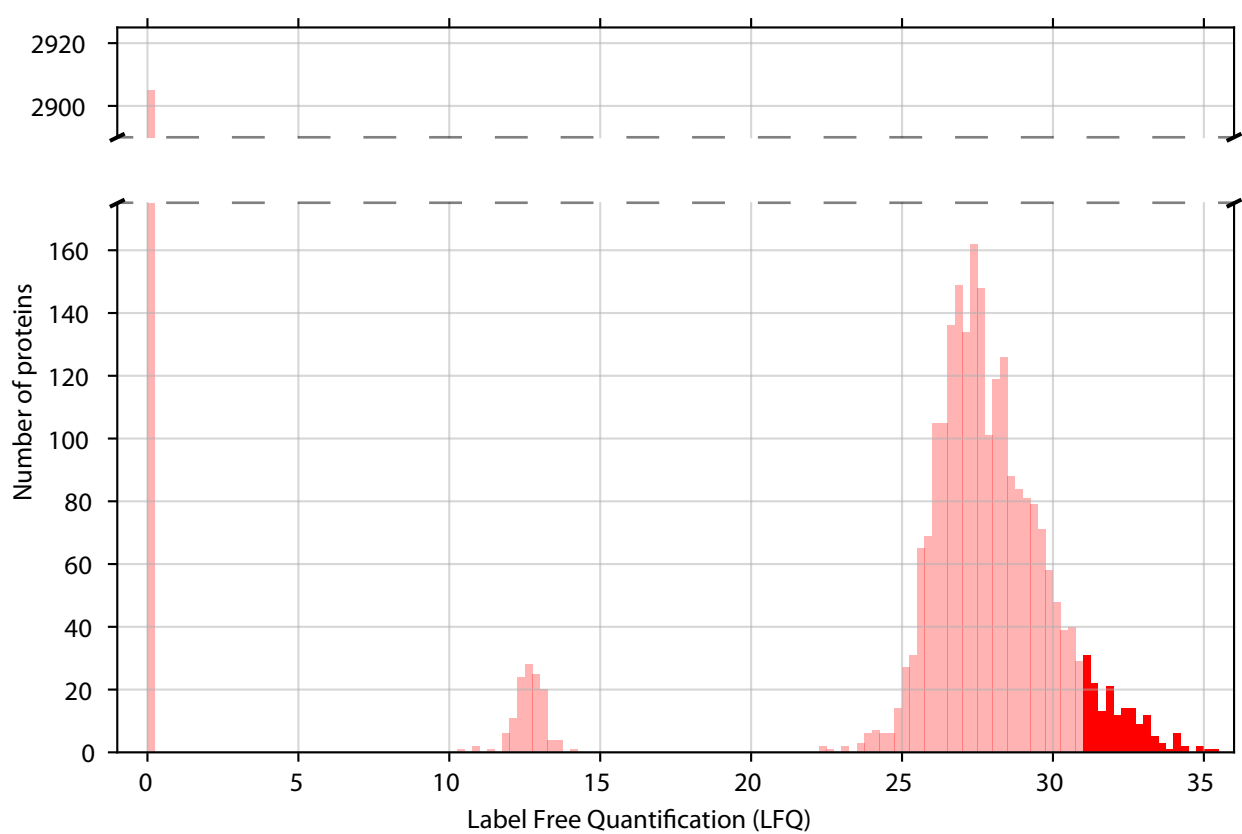

Supplement: Supplementary file 2 — Fig S2 [file JPI-70-e12713-s003.pdf]

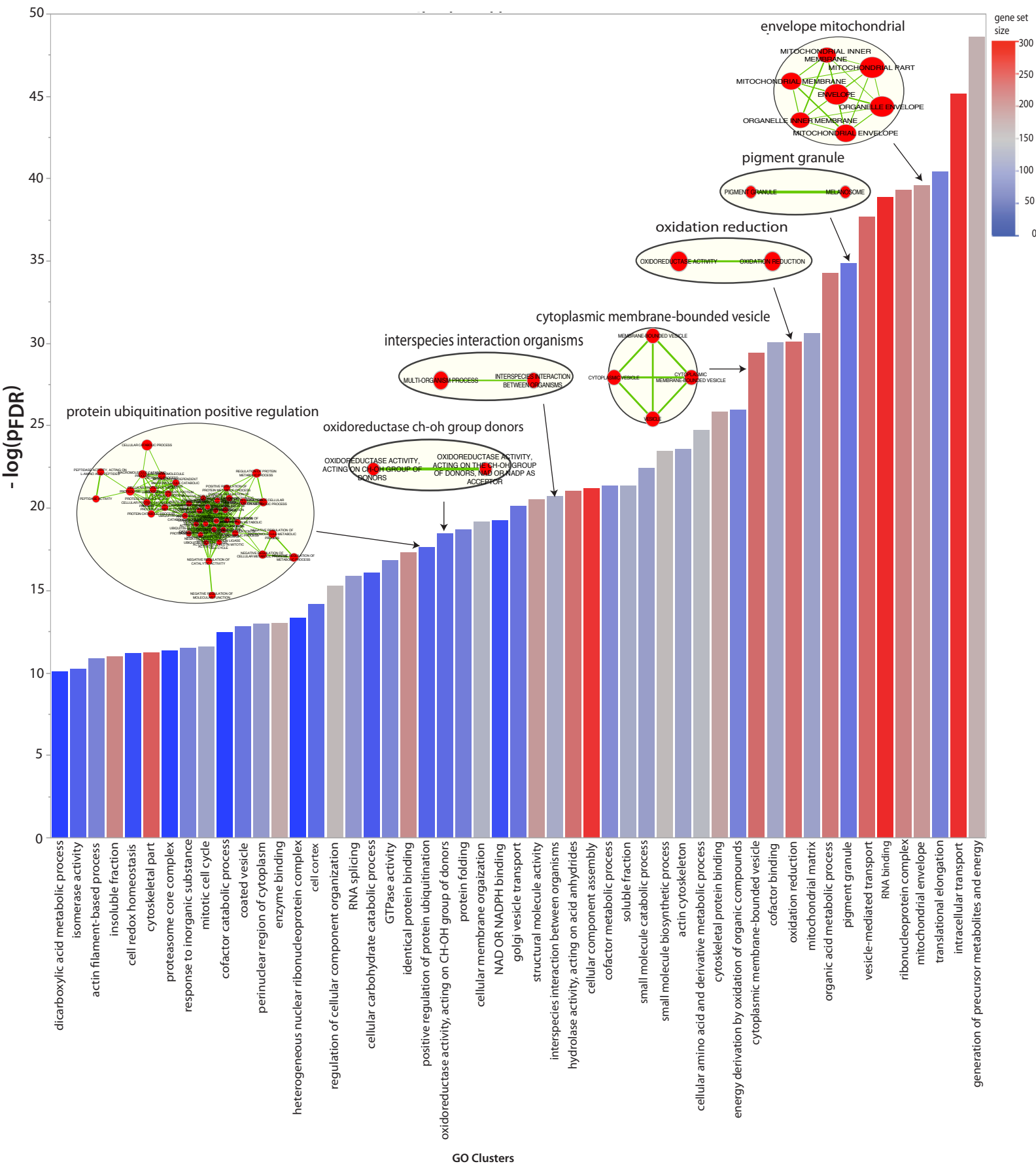

Supplement: Supplementary file 3 — Fig S3 [file JPI-70-e12713-s001.pdf]

# **AGRIN**

**A**

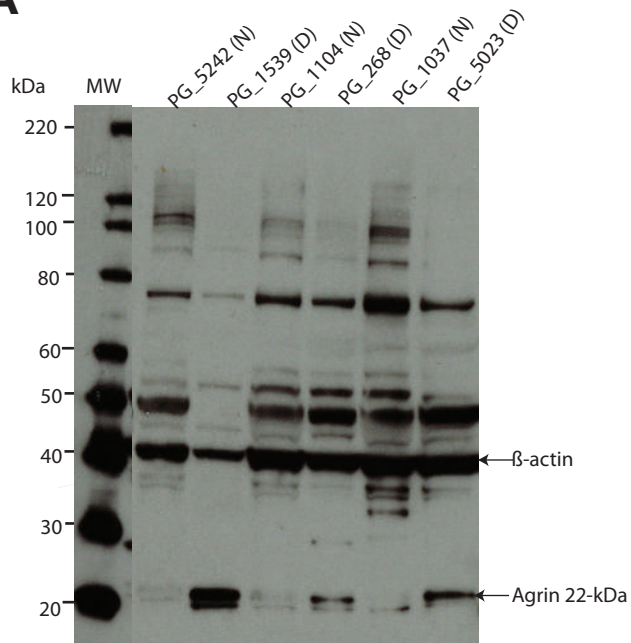

# **$\beta$ -2-Microglobulin**

**B**

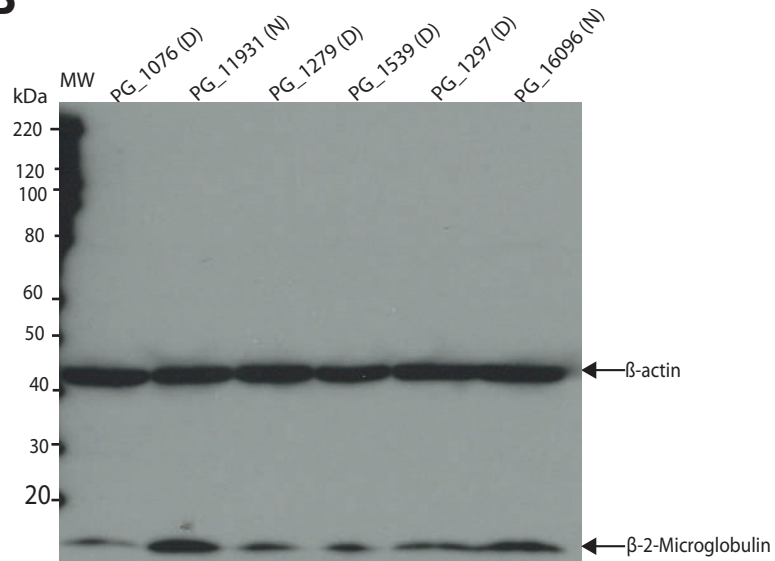

**C**

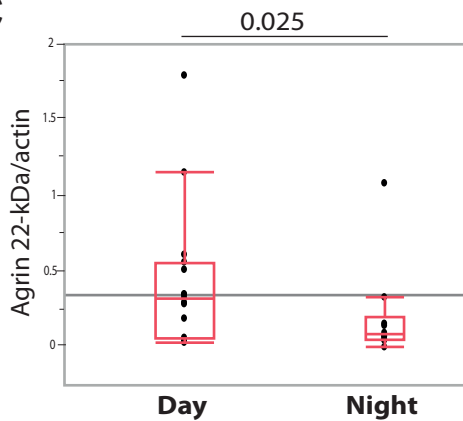

**D**

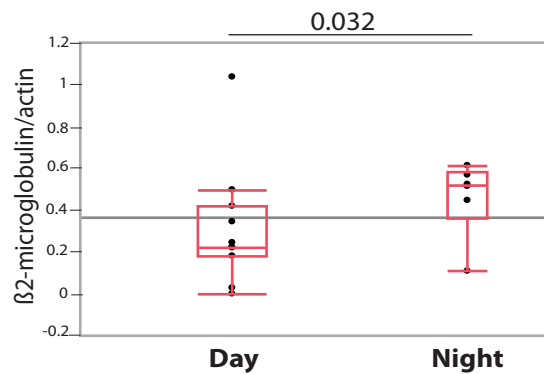

Supplement: Supplementary file 4 — Fig S5 [file JPI-70-e12713-s013.pdf]

# Autism vs Control

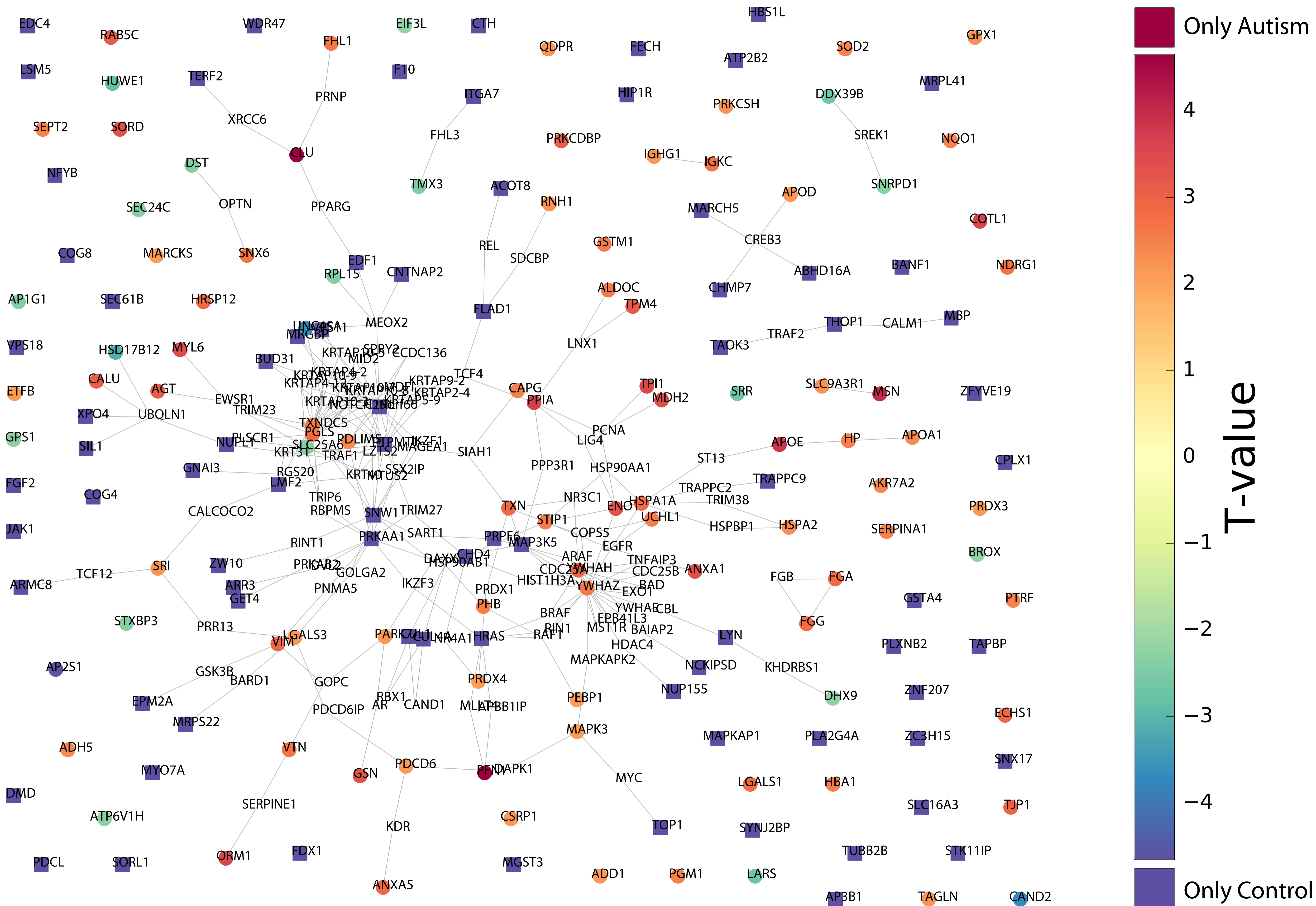

Supplement: Supplementary file 5 — Fig S6 [file JPI-70-e12713-s005.pdf]

YWHAE

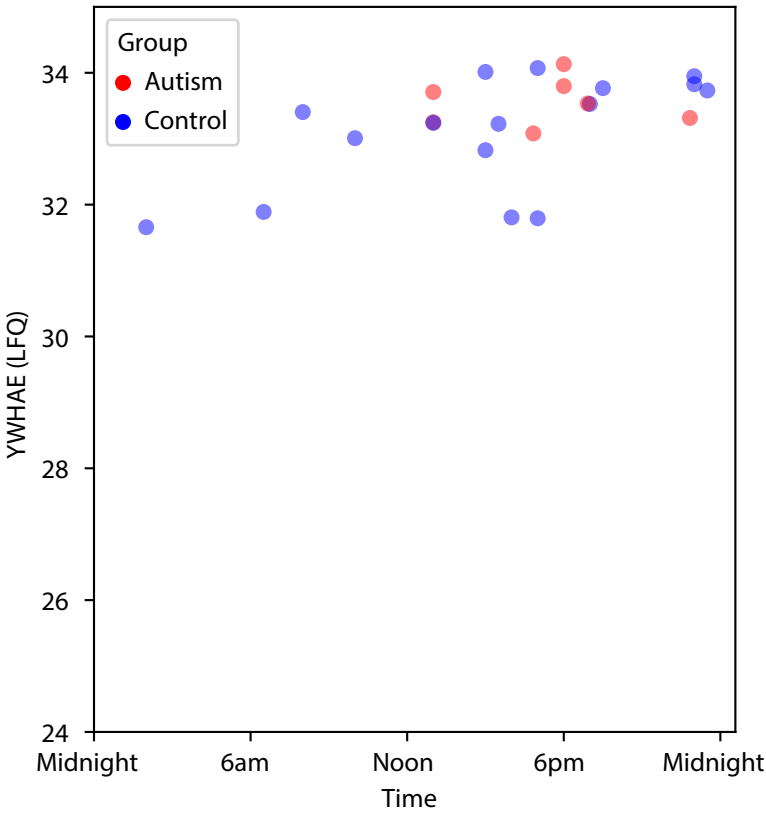

YWHAG

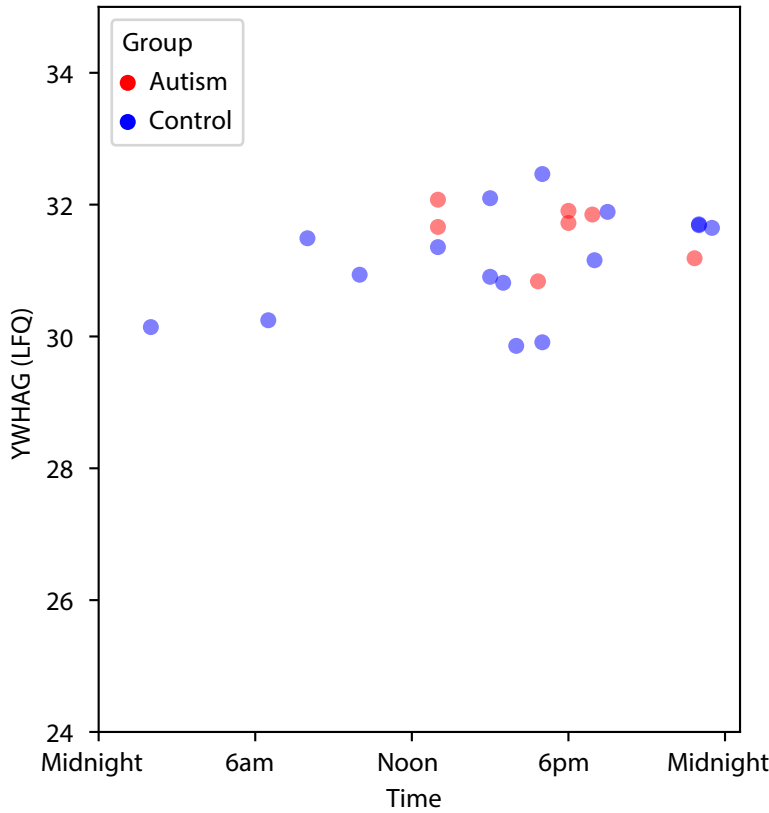

YWHAH

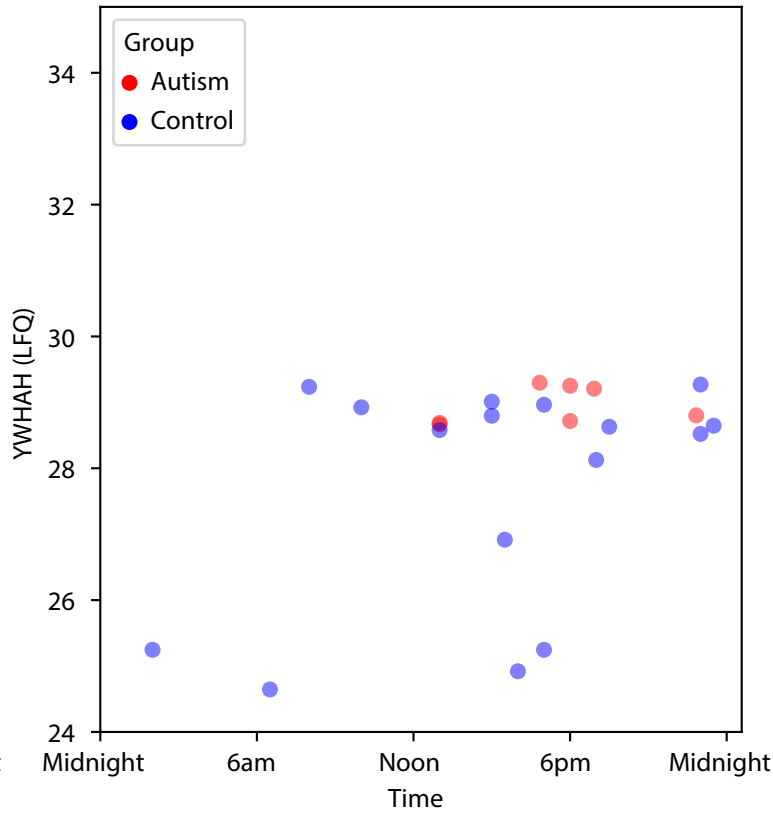

YWHAQ

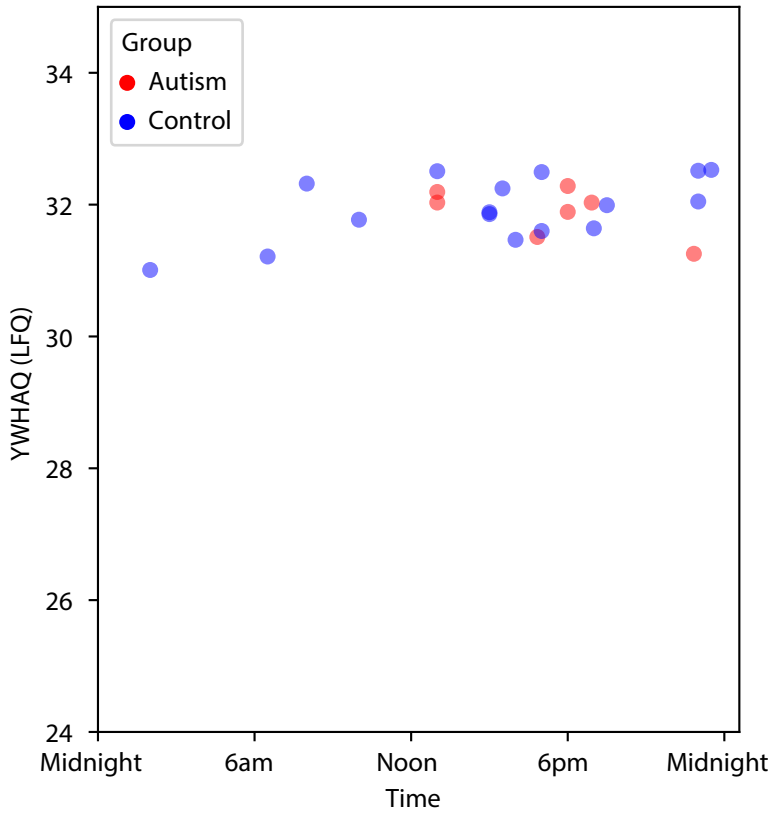

YWHAZ

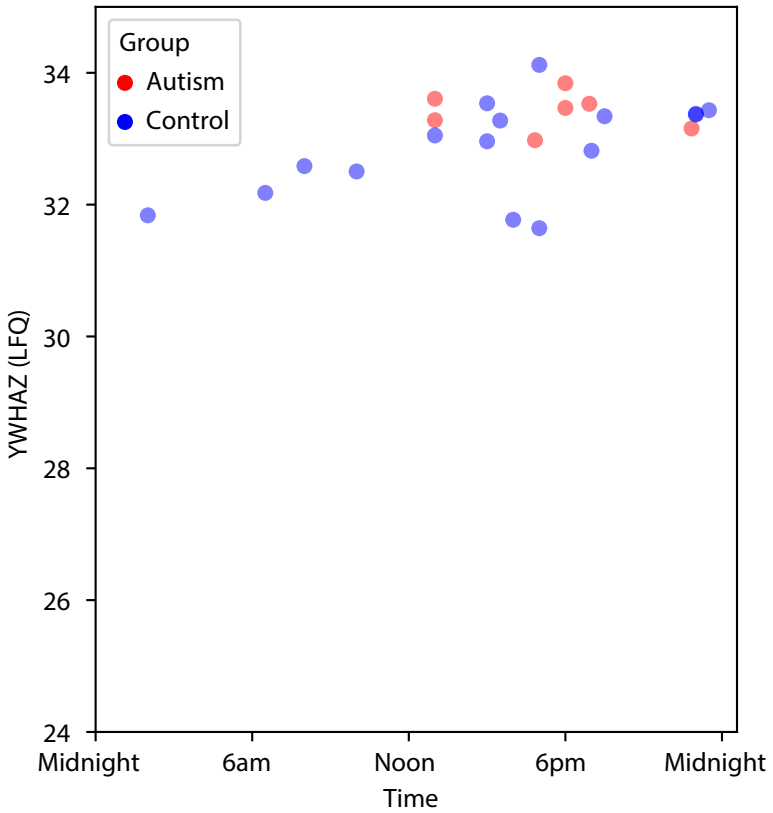

Supplement: Supplementary file 6 — Fig S7 [file JPI-70-e12713-s011.pdf]

**A**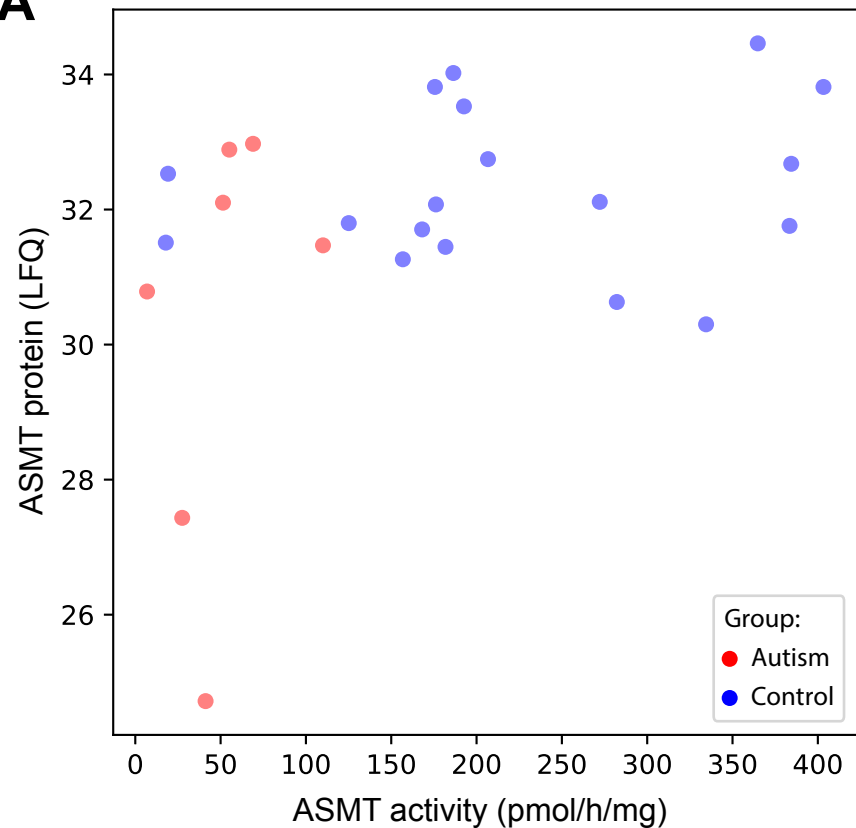**B**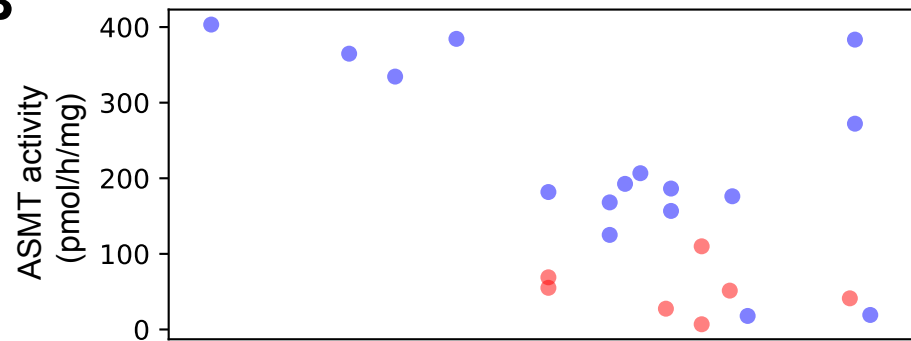**C**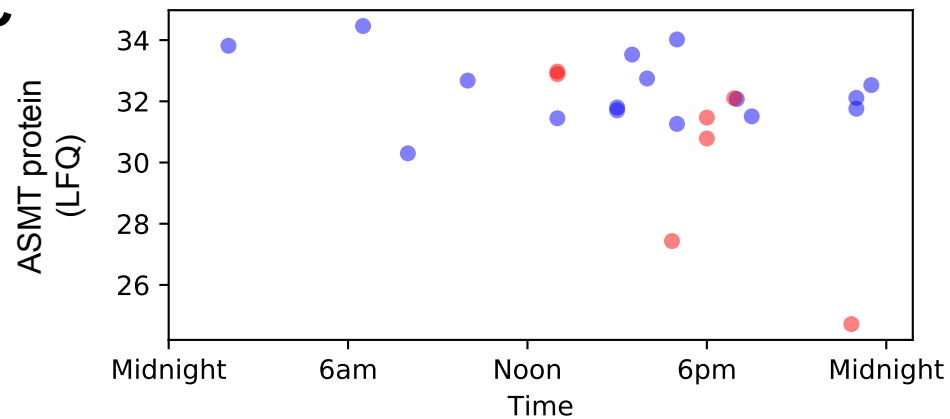

Supplement: Supplementary file 7 — Fig S8 [file JPI-70-e12713-s012.pdf]

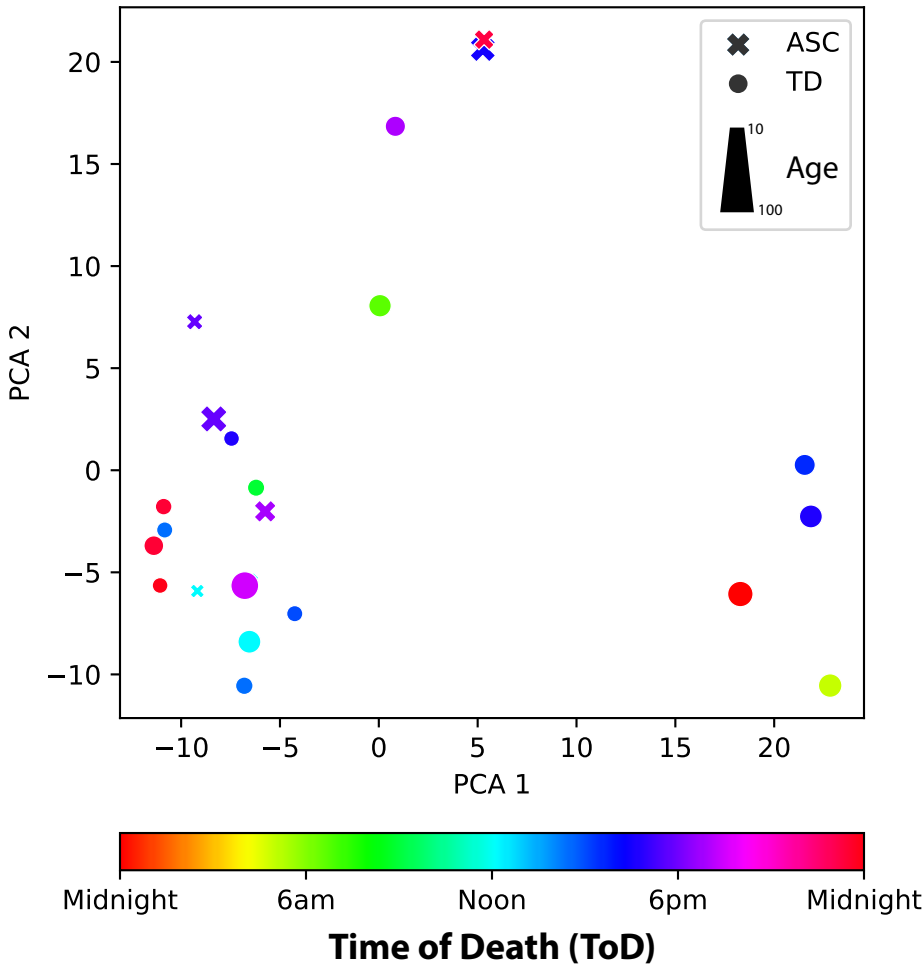

Supplement: Supplementary file 8 — Fig S9 [file JPI-70-e12713-s006.pdf]
