# Supplementary material for: Mass‐spectrometry analysis of the human pineal proteome during night and day and in autism
Source: J Pineal Res. 2021 Jan 11;70(3):e12713. doi: 10.1111/jpi.12713 (PMC8047921; doi:10.1111/jpi.12713)
Supplement: Supplementary file 14 — Supplementary Material [file JPI-70-e12713-s014.docx]

**Supporting Information**

**Title:** Mass-spectrometry analysis of the human pineal proteome during night and day and in autism.

**Authors:** Guillaume Dumas^1,2,*^, Hany Goubran-Botros^1,*^, Mariette Matondo^3,*^, Cécile Pagan^4,5^, Cyril Boulègue^3^, Thibault Chaze^3^, Julia Chamot-Rooke^3^, Erik Maronde^6,#^, Thomas Bourgeron^1,#^

**Affiliations:**

1. Human Genetics and Cognitive Functions, Institut Pasteur, UMR 3571 CNRS, University Paris Diderot, Paris, France.

2. Precision Psychiatry and Social Physiology laboratory, CHU Ste-Justine Research Center, Department of Psychiatry, University of Montreal, Quebec, Canada

3. Institut Pasteur, Unité de Spectrométrie de Masse pour la Biologie (MSBio), Centre de Ressources et Recherches Technologiques (C2RT), USR 2000 CNRS, Paris, France.

4. Paris Descartes University, Sorbonne Paris Cité, Paris, France.

5. Service de Biochimie et Biologie Moléculaire, INSERM U942, Hôpital Lariboisière, APHP, Paris, France.

6. Institute for Anatomy II, Faculty of Medicine, Goethe University, Frankfurt, Germany.
*,#. Authors contributed equally to this work.

**Correspondence :** Guillaume Dumas and Thomas Bourgeron, Human Genetics and Cognitive Functions, Institut Pasteur, 25 rue du Docteur Roux, 75015 Paris, France.

Email: [guillaume.dumas@centraliens.net](mailto:guillaume.dumas@centraliens.net) (G.D.); [thomasb@pasteur.fr](mailto:thomasb@pasteur.fr) (T.B.)

**Supplementary Figures**

**Supplementary Figure 1:** Global workflow of the analyses.

**Supplementary Figure 2:** Log-normalized Label Free Quantification (LFQ) values in all measured pineal samples from non-diagnosed control individuals.

**Supplementary Figure 3:** highly enriched pathways detected as Gene Ontology clusters identified with the EnrichmentMap algorithms.

**Supplementary Figure 4:** Characteristic profiles of rhythmic proteins expression with higher level during day (AGRN and IGHG2) or night (GFAP and CDH2).

**Supplementary Figure 5:** Subset of modulated proteins validated by Western blot with associated quantification.

**Supplementary Figure 6:** Interaction between the modulated proteins between Autism and Control groups.

**Supplementary Figure 7:** 14-3-3 proteins expression level across the day.

**Supplementary Figure 8:** ASMT enzyme activity and protein expression level with the variation across the day.

**Supplementary Figure 9:** PCA projection of all the pineal samples.

**Supplementary Tables** (available as Excel files)

**Supplementary Table 1:** Description of the samples.

**Supplementary Table 2:** Proteins LFQ and associated Z-score.

**Supplementary Table 3:** Enriched Gene Ontology pathways.

**Supplementary Table 4:** Proteins modulated between Night and Day.

**Supplementary Table 5:** Proteins modulated between Autism and Control groups.

**Supplementary Figure 1: Global workflow of the analyses. ASD : Autism Spectrum Disorders. BGLS: Bayesian Generalized Lomb-Scargle. JTK: Jonckheere-Terpstra-Kendall.**


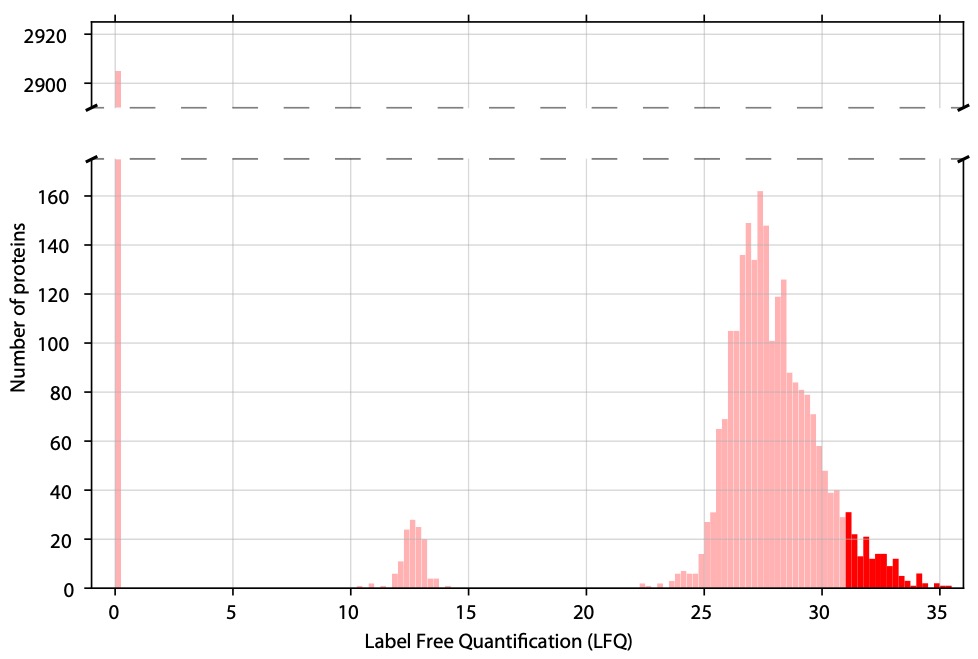


**Supplementary Figure 2: Log-normalized Label Free Quantification (LFQ) values in all measured pineal samples from non-diagnosed control individuals.**


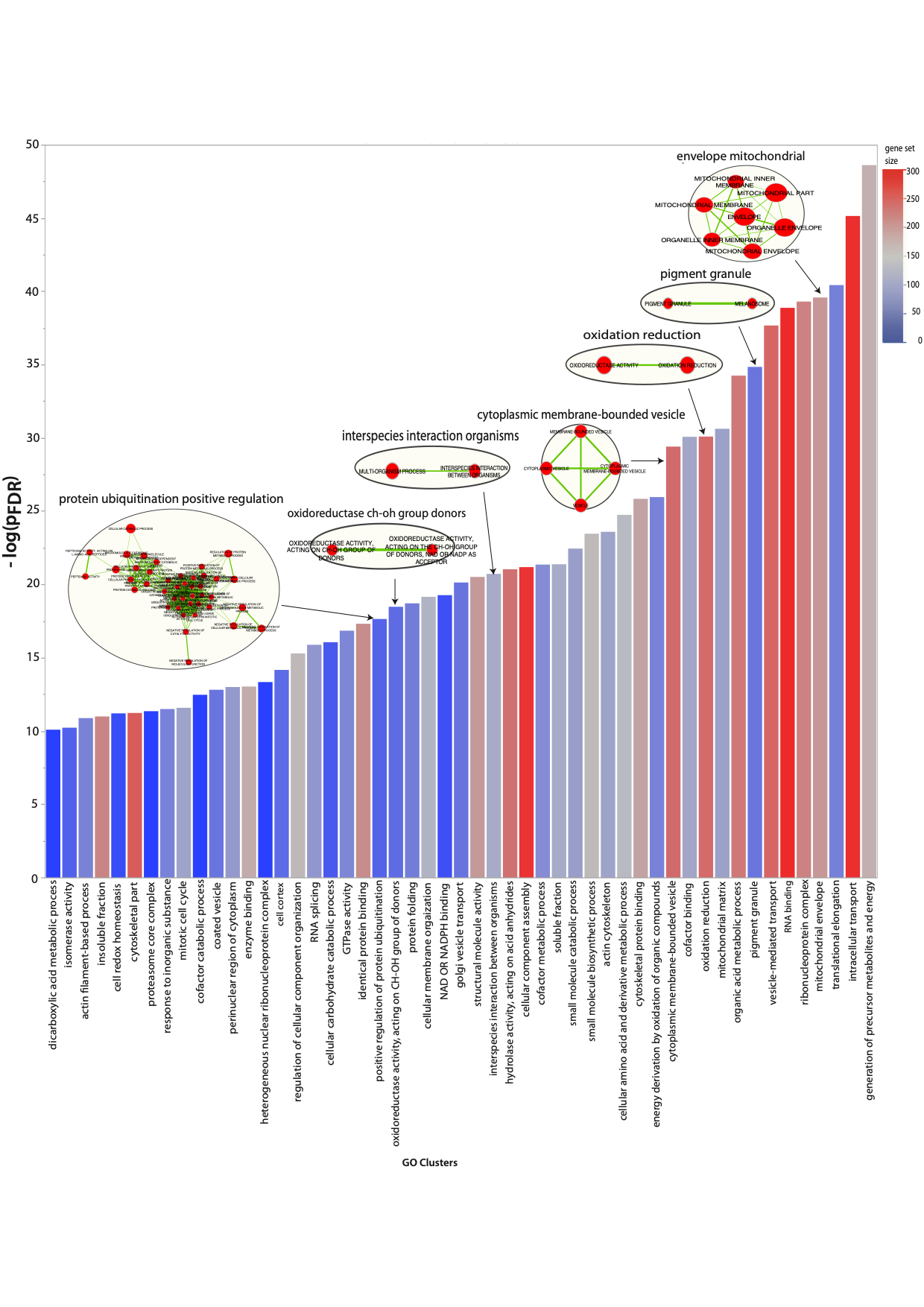
**Supplementary Figure 3: highly enriched pathways detected as Gene Ontology clusters identified with the EnrichmentMap algorithms.**

**Supplementary Figure 4:** **Characteristic profiles of rhythmic proteins expression with higher level during day (AGRN and IGHG2) or night (GFAP and CDH2).**

**
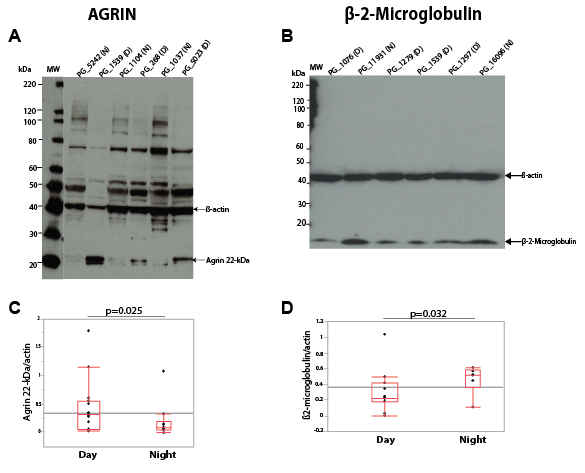
**

**Supplementary Figure 5: Subset of modulated proteins validated by Western blot (A,B) with associated quantification (C,D). Box-plots indicates the quartiles of the data, with middle line indicating the median.**

**
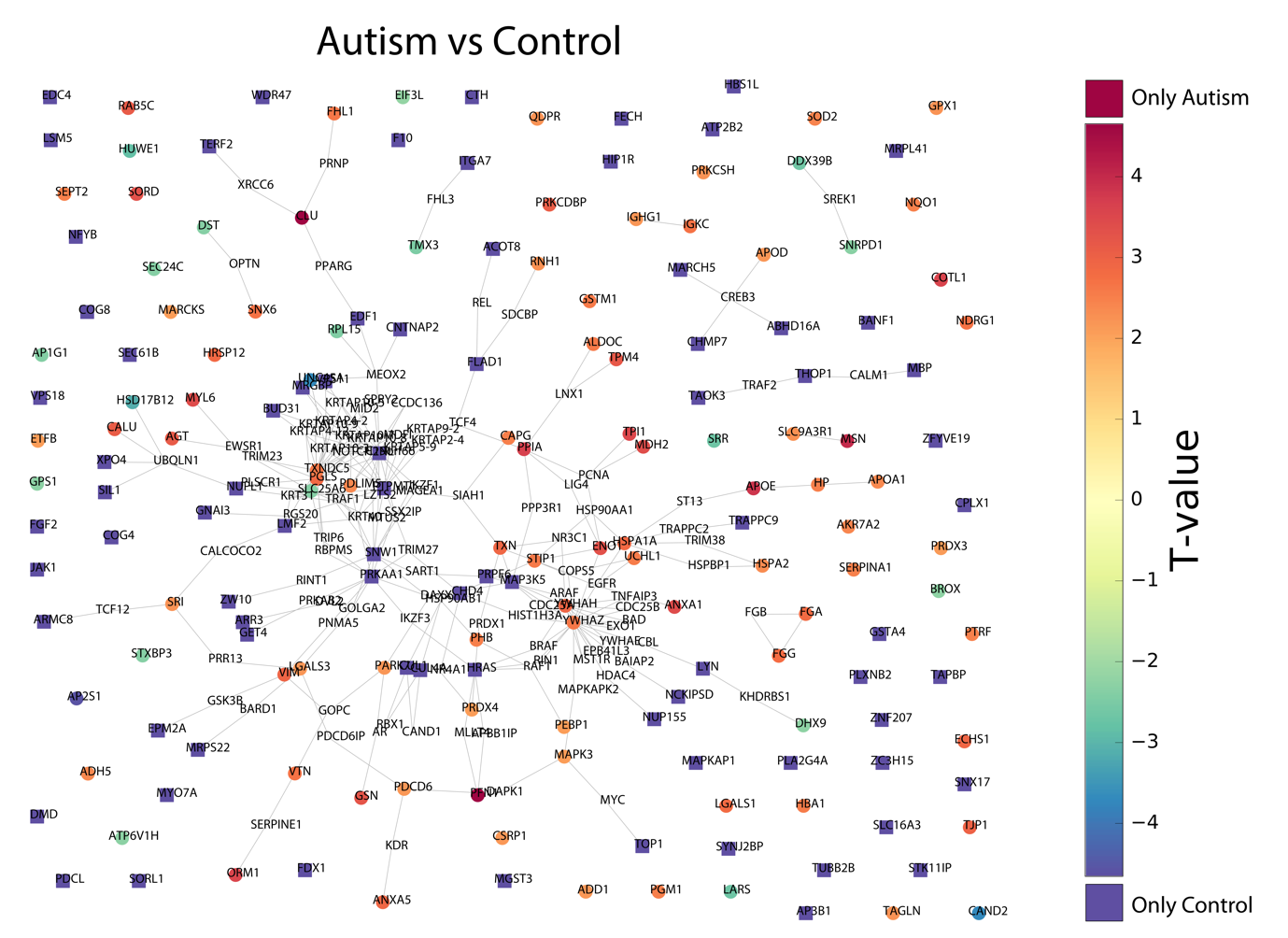
Supplementary Figure 6: Interaction between the modulated proteins between Autism and Control groups.**

**
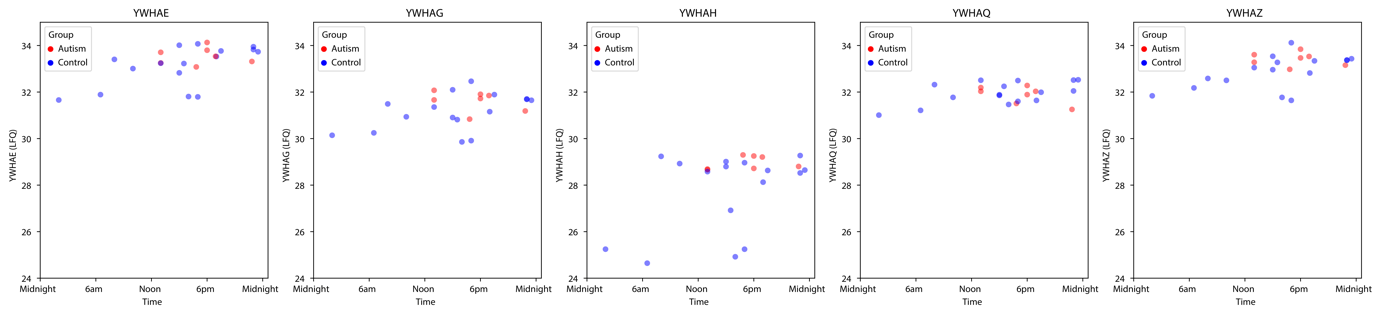
**

**Supplementary Figure 7:** **14-3-3 proteins expression level across the day.** Autism and Control groups are respectively in red and blue.

**
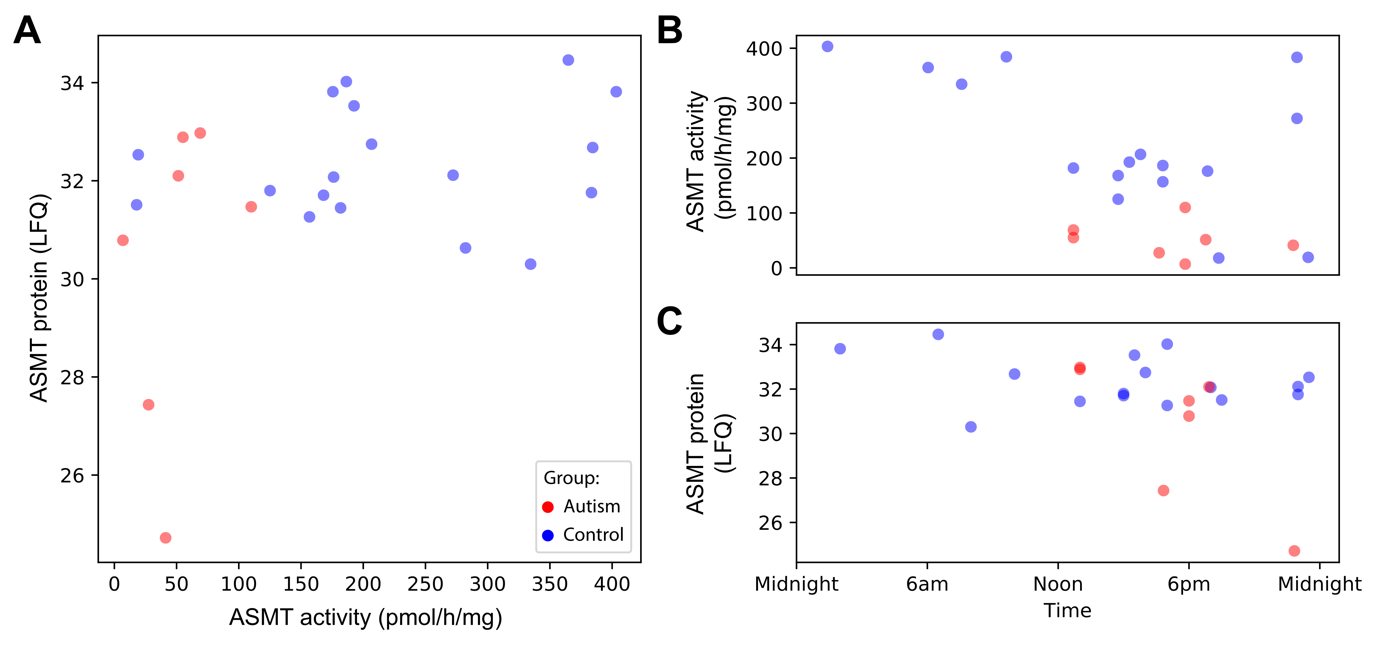
**

**Supplementary Figure 8:** **ASMT enzyme activity and protein expression level (A) with the variation across the day (B,C).** Autism and Control groups are respectively in red and blue.

**
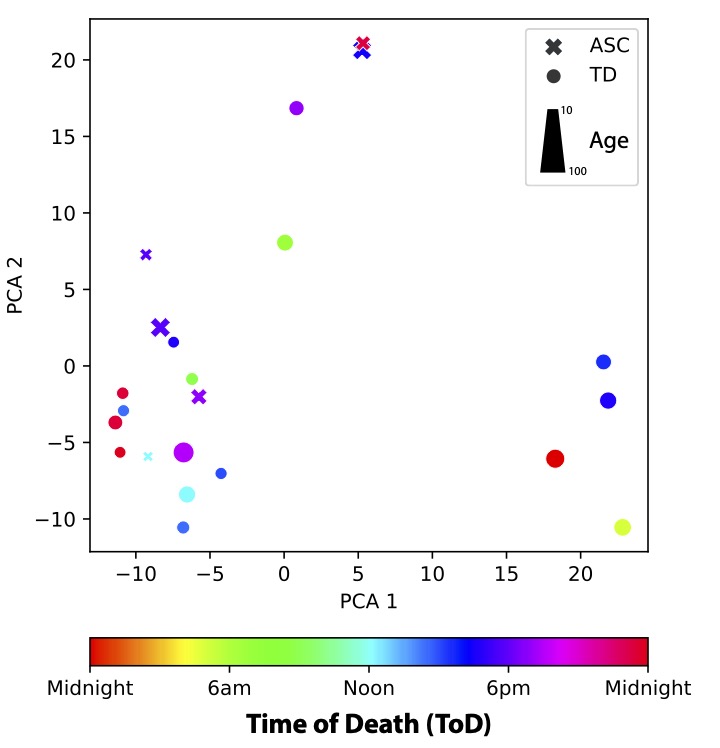
**

**Supplementary Figure 9:** **PCA projection of all the pineal samples.** Color code for the time of death; Size for the age; and Marker type for the status of the participant (Autism Spectrum Condition: cross; Typically Developing: circle).
